# Long-term dynamics of aberrant neuronal activity in awake Alzheimer’s disease transgenic mice

**DOI:** 10.1038/s42003-021-02884-7

**Published:** 2021-12-07

**Authors:** V. Korzhova, P. Marinković, J. Rudan Njavro, P. M. Goltstein, F. Sun, S. Tahirovic, J. Herms, S. Liebscher

**Affiliations:** 1 German Center for Neurodegenerative Diseases (DZNE), 81377 Munich, Germany; 2 Center for Neuropathology and Prion Research, Ludwig-Maximilians University Munich, 81377 Munich, Germany; 3 Munich Cluster for Systems Neurology (SyNergy), 81377 Munich, Germany; 4 Institute of Clinical Neuroimmunology, Klinikum der Universität München, Ludwig-Maximilians University, 82152 Martinsried, Germany; 5 Max Planck Institute of Neurobiology, 82152 Martinsried, Germany; 6 Biomedical Center, Medical Faculty, Ludwig-Maximilians University Munich, 82152 Martinsried, Germany

**Keywords:** Alzheimer's disease, Alzheimer's disease

## Abstract

Alzheimer’s disease (AD) is associated with aberrant neuronal activity, which is believed to critically determine disease symptoms. How these activity alterations emerge, how stable they are over time, and whether cellular activity dynamics are affected by the amyloid plaque pathology remains incompletely understood. We here repeatedly recorded the activity from identified neurons in cortex of awake APPPS1 transgenic mice over four weeks during the early phase of plaque deposition using in vivo two-photon calcium imaging. We found that aberrant activity during this stage largely persisted over the observation time. Novel highly active neurons slowly emerged from former intermediately active neurons. Furthermore, activity fluctuations were independent of plaque proximity, but aberrant activity was more likely to persist close to plaques. These results support the notion that neuronal network pathology observed in models of cerebral amyloidosis is the consequence of persistent single cell aberrant neuronal activity, a finding of potential diagnostic and therapeutic relevance for AD.

## Abstract (web summary)

To advance our understanding of aberrant neuronal activity in Alzheimer’s disease (AD) Korzhova et al. use in vivo two-photon calcium imaging to record activity from cortical neurons of awake APPPS1 transgenic mice over four weeks, during the early phase of plaque deposition. They demonstrate that neuronal network pathology in models of cerebral amyloidosis is the consequence of persistent single cell aberrant neuronal activity, which could have diagnostic and therapeutic relevance for AD.

## Introduction

Alzheimer’s disease (AD), the most common form of dementia, is histopathologically characterized by the accumulation of diverse assemblies of the amyloid–beta peptide (Aβ) within the CNS, which are accompanied by aberrant neuronal hyper- and hypoactivity^1,2^, alterations in oscillatory activity and network hypersynchrony^2^. These aberrant neuronal activity levels and network dysfunction were shown to be one of the very early events of a pathogenic cascade in AD and to determine the level of cognitive impairment in affected individuals^2–4^. Single cell aberrant activity, in particular ‘hyperactive’ cells, have to date been mainly characterized in vivo in anesthetized mice^1,5–7^, while recent studies conducted in awake mice did not report the occurrence of such phenomena^8–10^. It thus remains open whether hyperactivity at the single cell level is present during wakefulness in AD models and most importantly, what their long-term dynamics in vivo are. Hyper- and hypoactivity of individual neurons could either be a transient phenomenon or, alternatively, represent a stable feature, present over prolonged periods of time up to weeks or months. Both scenarios are conceivable, given the different mechanisms proposed to underlie the development of these activity changes. Oligomeric Aβ has been suggested to directly or indirectly affect synaptic function. As such, Aβ was shown to bind to several surface molecules of synapses^11,12^, particularly to postsynaptic elements of excitatory synapses^13^. Moreover, Aβ causes synaptic instability, a shrinkage of dendritic spines and synaptic loss, which are accompanied by a reduction in LTP and increased LTD, seen in vitro and in vivo^14–16^. Somewhat counterintuitively, Aβ can trigger an ‘aberrant’ increase in neuronal activity, observed upon acute application^17^, as well as under chronic conditions, such as in transgenic mouse models. This aberrant activity gain has been suggested to result from the activation of NMDA and AMPA receptors^1,17–19^, through increased glutamate release probability^20^, blockage of glutamate uptake^7,21^, or dysfunction of presynaptic intracellular Ca^2+^ stores^22^. Notably, recent evidence suggests that various neuronal cell-types are affected differentially in the disease. As such, dysfunction of certain types of interneurons, e.g., parvalbumin -, somatostatin - or vasoactive intestinal peptide expressing interneurons, has been reported^4,23,24^. These cell-type specific impairments are likely to cause a deficit in the otherwise tightly regulated balance between excitation and inhibition (E/I), leading to more global, circuit-level defects in AD. E/I imbalance could, therefore, represent a main driver of aberrant activity in AD, causing a more stable, slowly progressing change in neuronal activity levels. To address the question of whether aberrant activity levels exist during wakefulness and whether they represent a stable or transient trait of neurons, we monitored the activity of individual identified neurons in cortex of awake APPPS1 transgenic mice and their non-transgenic littermates over four weeks by means of in vivo two-photon calcium imaging. Our data show that (a) hyperactivity is present in ***awake*** AD transgenic mice, (b) these highly active neurons develop slowly from former intermediately active neurons, (c) that activity levels largely persist over the four weeks investigational period in the early phase of the disease in the APPPS1 mouse model, and (d) that activity fluctuations are independent of amyloid plaque proximity.

## Results

**Fig. 1:**
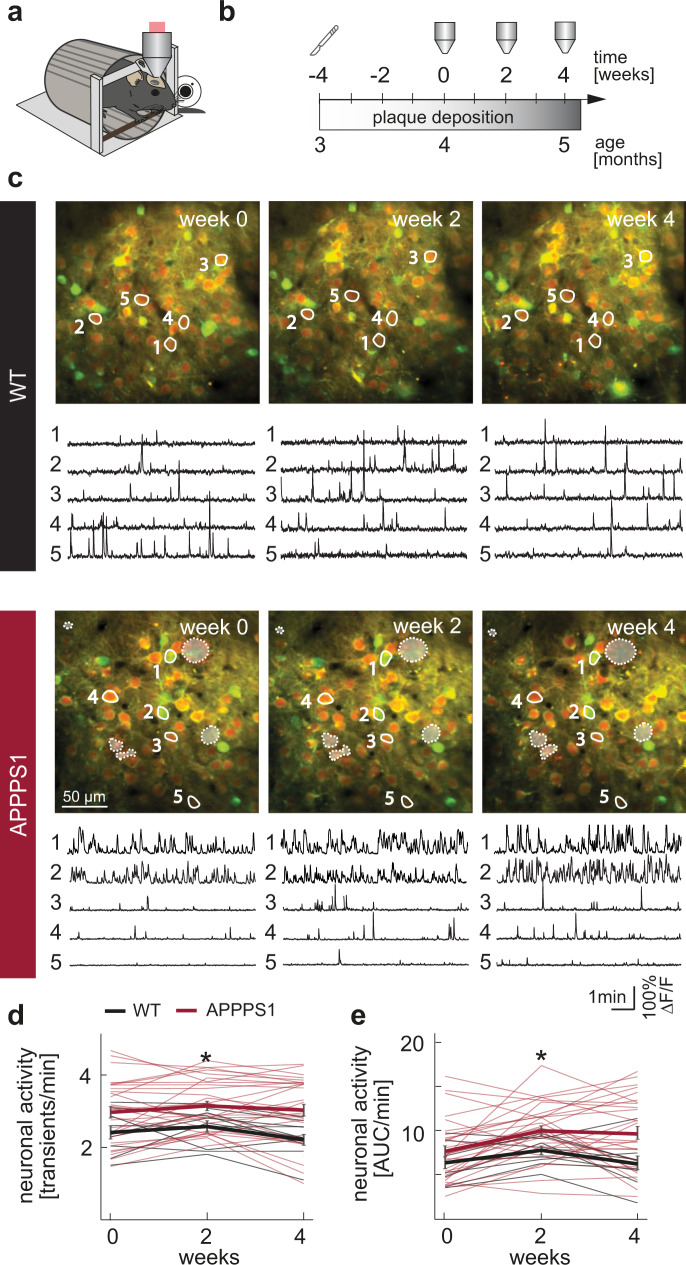
Chronic in vivo imaging of individual neurons in awake mice. **a** Schematic of the in vivo imaging setup. Mice were head-fixed, while neuronal activity was recorded through a cranial window. Whisking was recorded by a web camera. **b** Experimental timeline. Window implantation (scalpel icon) was conducted four weeks prior to the first imaging session. Imaging (objective icon) was performed at two-week intervals. Plaque deposition in the APPPS1 mouse line starts at ~8 weeks of age and plaque growth (gray gradient) takes place throughout the lifetime of these mice. **c** Representative projections of field of views (FOVs) in a WT and an APPPS1 mouse (red: mRuby2 expression, green: GCaMP6s, dashed lines indicate the location of amyloid plaques as assessed in larger overview stacks). Example calcium traces of neurons labelled within the projections are shown below. **d** Average neuronal activity of neurons in WT and APPPS1 mice, measured as transients per minute (effect of group: *F*_1,72_ = 6.07, *p* = 0.02; effect of time: *F*_2,72_ = 2.23, *p* = 0.12; group-by-time interaction effect: *F*_2,72_ = 0.91, *p* = 0.41, two way repeated measures ANOVA; thin lines: averages of individual FOVs (same set of neurons imaged over three consecutive time points); thick lines: mean ± SEM for each time point). **e** Neuronal activity assessed by quantification of  the area under the curve (AUC) per minute across all time points (*F*_1,72_ = 4.58, *p* = 0.04; effect of time: *F*_2,72_ = 9.72, *p* = 0.0002; group-by-time interaction effect: *F*_2,72_ = 1.67, *p* = 0.2, two way repeated measures ANOVA; WT *n* = 9 (5 mice), APPPS1 *n* = 29 experiments (9 mice); thick lines: mean ± SEM) is increased in APPPS1 mice. *p < 0.05.

To address the question whether neuronal activity is altered in ***awake*** APPPS1 mice and if so, how stable these aberrant activity levels are, we longitudinally monitored the activity of the same identified neurons in layer 2/3 of the frontal cortex over four consecutive weeks. We performed calcium imaging, using the genetically encoded calcium indicator GCaMP6s^25,26^ in awake Amyloid Precursor Protein – Presenilin 1 (APPPS1) transgenic mice^27^. We measured neural activity in three consecutive imaging sessions, spaced by two weeks (Fig. 1a–c, Supplementary Fig. 1). We started our recordings at the age of 4 months, which in this mouse model represents the early phase of Aβ plaque deposition^28^. To characterize the cell identity of the imaged neurons, we performed post-hoc immunostainings and found that the vast majority (96.4%) of GCaMP6s expressing cells was, in fact, excitatory (Supplementary Fig. 2a, b). We first compared the average spontaneous neuronal activity of layer 2/3 neurons in frontal cortex of APPPS1 mice to their WT control littermates in awake, head-fixed mice, trained to sit quietly in a restrainer. Our investigations revealed that both the frequency of transients (Fig. 1d), as well as the area under the curve of the calcium signal, measured as an integral of the ΔF/F trace over time (Fig. 1e, Supplementary Data 1), were significantly increased in APPPS1 mice throughout the experimental period (both at the level of individual experiments, as well as of individual mice (Supplementary Fig. 2c, d)). We also observed increased neuronal activity levels associated with whisking in APPPS1 mice (Supplementary Fig. 2e, f). Overall, the activity of individual neurons during quiet wakefulness was highly correlated with the activity levels measured during whisking (Supplementary Fig. 2g, h).

### Activity change over time

**Fig. 2:**
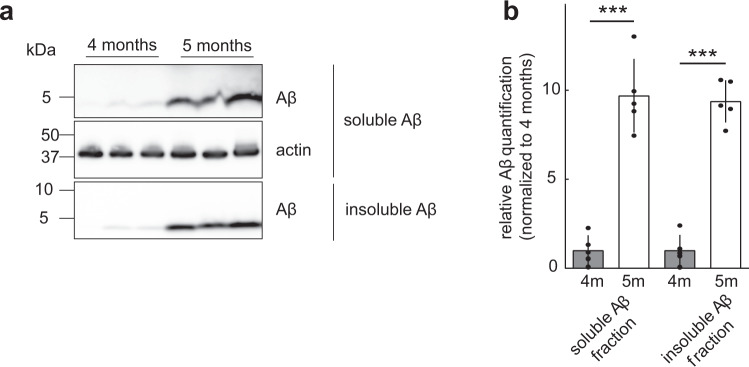
Aβ load in 4 and 5 months old APPPS1 mice. **a** Western blot analysis of soluble (STET, *p* < 0.001) and insoluble (FA, *p* < 0.001, unpaired two-tailed Student’s *t* test) Aβ fractions of APPPS1 mice. **b** Quantification of Aβ Western blot signals demonstrates a significant increase of Aβ load from 4 to 5 months of age (9.7- and 9.4-fold in the soluble and insoluble fraction, respectively,). Values are normalized to the values of Aβ at 4 months and represent the mean ± SEM. ***p < 0.001.

**Fig. 3:**
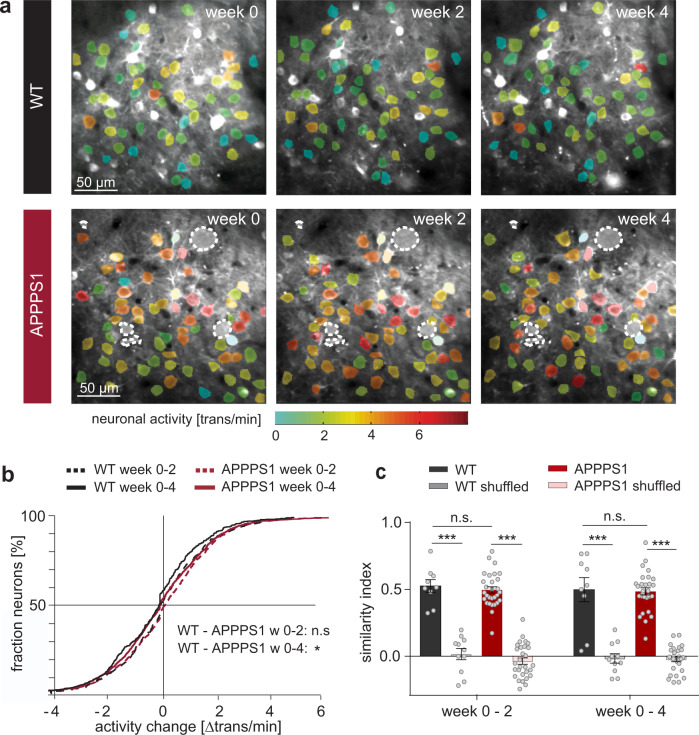
Neuronal activity levels are largely stable over time. **a** Representative FOVs across all 3 time points from a WT and APPPS1 mouse, superimposed by the ROI (region of interest) selection mask (color codes for neuronal activity (transients per minute); filled cells were not included in the analysis; dashed lines: amyloid plaques). **b** Change in neuronal activity (week 0–2: *p* = 0.08, week 0–4: *p* = 0.03, KS test, WT *n* = 471 neurons (5 mice), APPPS1 *n* = 1514 neurons (9 mice)). **c** Similarity of neuronal activity within a given field of view (week 0–2: *F*_3,74_ = 105.7, *p* < 10^−4^, one way ANOVA, WT vs WT shuffled *p* < 10^−4^, WT vs APPPS1 *p* = 0.9, APPPS1 vs APPPS1 shuffled *p* < 10^−4^, Bonferroni’s *p*ost-hoc test; week 0–4: *F*_3,74_ = 78.51, *p* < 10^−4^, one way ANOVA, WT vs WT shuffled *p* < 10^−4^, WT vs APPPS1 *p* = 0.9, APPPS1 vs APPPS1 shuffled *p* < 10^−4^, Bonferroni’s post-hoc test). Data are mean ± SEM. *p < 0.05, ***p < 0.001.

During this early stage of the disease ^27^, we found a strong increase in soluble and insoluble Aβ in the brains of APPPS1 mice between the age of 4 to 5 months  (Fig. 2a, b, Supplementary Data 2, Supplementary Fig. 3) and asked how it would affect the activity levels of individual neurons. To address this question, we compared the distribution of the changes in activity of individual neurons assessed in awake mice between time points (Fig. 3a, b, Supplementary Data 3). Interestingly, the distribution of those activity changes did not differ between WT and APPPS1 mice between week 0 and 2 and was only slightly altered from week 0 to 4 (Fig. 3b). In addition, we computed the similarity of activity levels of individual neurons within a given field of view across time points (Supplementary Fig. 4) and did not observe a difference between WT and APPPS1 mice (Fig. 3c, Supplementary Data 4). To mimic a rapid and transient change in activity levels, we shuffled the data set by randomly changing the order of neurons at time point week 2 and 4 compared to week 0. The similarity indexes of the shuffled data were close to zero and thus significantly lower than the similarity observed within the actual data set (Fig. 3c). Together, these findings indicate that overall activity levels remain largely constant over time with only minor fluctuations in activity (±2 transients/min for >75% of all neurons). Aberrant activity is thus a persistent single cell feature over at least four weeks in awake APPPS1 transgenic mice.

### Emergence and fate of highly active cells

**Fig. 4:**
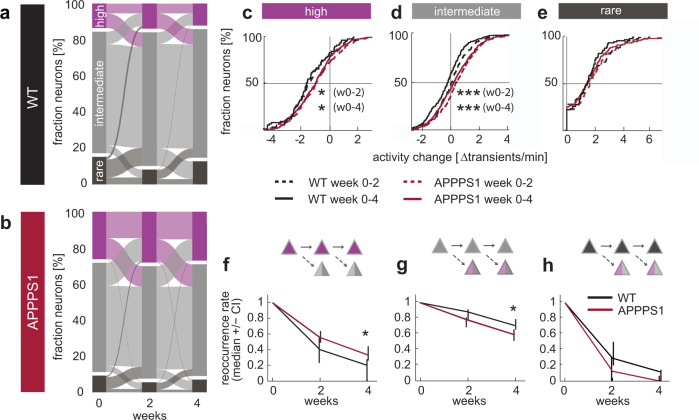
Category-specific changes in neuronal activity. **a**, **b** Alluvial plots depicting the fractional change between time points of highly (>4 transients/min), intermediately (0.25–4 transients/min) and rarely active (<0.25 transients/min) neurons in WT (**a**) and APPPS1 (**b**) mice over time (fraction highly active neurons: week 0–4 effect of group: *F*_1,72_ = 6.96, *p* = 0.012, effect of time: *F*_2,72_ = 0.15, *p* = 0.86, group-by-time interaction effect: *F*_2,72_ = 0.83, *p* = 0.44; intermediately active cells: week 0–4 effect of group: *F*_1,72_ = 5.52, *p* = 0.024, effect of time: *F*_2,72_ = 3.47, *p* = 0.036, group-by-time interaction effect: *F*_2,72_ = 0.006, *p* = 0.45; rarely active cells week 0–4 effect of group: *F*_1,72_ = 3.9, *p* = 0.056, effect of time: *F*_2,72_ = 5.24, *p* = 0.0075, group-by-time interaction effect: *F*_2,72_ = 0.51, *p* = 0.6, WT *n* = 9 (5 mice), APPPS1 *n* = 29 experiments (9 mice), two-way repeated measures ANOVA). **c**–**e** Activity-category specific changes in neuronal activity throughout the 4-week investigation period. **c** Highly active neurons in APPPS1 mice underwent a smaller reduction in activity than those neurons in their WT littermates (*p* = 0.046 for week 0–2; *p* = 0.012 for week 0–4, KS test, WT *n* = 81 neurons, APPPS1 *n* = 444 highly active neurons). **d** Intermediately active neurons increased their activity on average in APPPS1 compared to WT (*p* < 10^−4^ for week 0–2; *p* < 10^−7^ for week 0–4, KS test, WT *n* = 309 neurons, APPPS1 *n* = 951 intermediately active neurons). **e** Activity change of rarely active neurons did not differ between APPPS1 and WT mice (*p* = 0.77 week 0–2; *p* = 0.24 week 0–4, KS test, WT *n* = 94 neurons, APPPS1 *n* = 177 rarely active neurons). **f** Reoccurrence rate over four weeks of highly (*p* = 0.025), **g** intermediately and (*p* = 0.019) **h** of rarely active neurons (*p* = 0.1, all Mann Whitney U test, data are median ± 95% CI), *P < 0.05, **P < 0.01, ***P < 0.001.

We next asked whether the change in activity hinges on the activity history. To this end, we classified neurons based on their initial activity levels into highly (>4 transients/min), intermediately (0.25–4 transients/min) and rarely (<0.25 transients/min) active neurons. In agreement with previous studies, conducted in anesthetized mice^1,5^, we found that also awake APPPS1 mice had a significantly larger fraction of highly active neurons, while intermediately active cells were less abundant in APPPS1 mice (Fig. 4a, b, Supplementary Data 5 and 6). The fraction of rarely active cells, however, was not significantly affected in APPPS1 mice (Fig. 4a, b). Despite smaller, but balanced fluctuations, the fractions of the different activity categories remained largely constant in both genotypes throughout the four-week imaging period. A category-specific investigation, however, revealed differences. Highly active neurons in APPPS1 mice underwent a lower reduction in activity than their counterparts in WT mice (Fig. 4c, Supplementary Data 7). Intermediately active neurons in APPPS1 mice, on the other hand, displayed a small increase in activity over time compared to intermediately active neurons in WT mice (Fig. 4d, Supplementary Data 8). Changes in the activity of rarely active neurons, however, did not differ between genotypes (Fig. 4e, Supplementary Data 9). As the absolute change in activity is affecting the likelihood to remain within a given category, we next compared the stable fraction of neurons within each activity category over time (reoccurrence rate, Fig. 4f–h, Supplementary Data 10). In accordance with the change in activity levels, we found that highly active neurons were significantly more likely to remain within the same category in APPPS1 mice (Fig. 4f), while intermediately active neurons were less likely to stay intermediately active over four weeks in APPPS1 mice (Fig. 4g). The fraction of consistently rarely active neurons after four weeks was close to zero and not different between genotypes (Fig. 4h). The overall stability of the three activity categories throughout the four-week observation time is based on a largely balanced fractional loss and gain of neurons within each category (see Supplementary Fig. 5a–c).

Aberrantly highly active (hyperactive) neurons are considered a key pathophysiological feature of mouse models of cerebral amyloidosis^1^. How hyperactivity emerges, however, remains elusive. Does the activity of certain neurons slowly increase over time, or is there a sudden pronounced increase in activity detectable in some neurons? To address this question, we analyzed the population of neurons that only turned highly active at week 2 and 4, respectively (Supplementary Fig. 5d–g). These ‘novel highly active’ neurons were almost exclusively recruited from former intermediately active neurons in both WT and APPPS1 mice (week 2: WT 79% and APPPS1 94% former intermediate, week 4: WT 96% and APPPS1 97% former intermediately active neurons, Fig. 4a, b). To next quantify the rate of this activity shift we computed the fraction of intermediately active neurons, which had transitioned into highly active neurons at the subsequent time point. While from week 0–2 in WT mice 9.7 % of the intermediately active neurons transitioned into highly active, the same was true for 21% of intermediately active cells in APPPS1 mice (Supplementary Fig. 5d). Those novel highly active neurons at week 2 gained on average 2.48 (1.94–3.09 CI) transients/min in WT and 2.03 (1.91–2.2 CI) transients/min in APPPS1 mice (Supplementary Fig. 5e). Similar changes were observed between week 2–4 (Supplementary Fig. 5f, g) with 7.3% (week 2–4) of intermediately active cells transitioning into highly active neurons in WT and 18.4% (week 2–4) of intermediately active neurons in APPPS1 mice (Supplementary Fig. 5f). Again, no difference in overall activity gain was detected for those intermediately active cells becoming highly active (Supplementary Fig. 5g). At week 2, however, the neuronal activity of intermediately active cells was already significantly higher in APPPS1 mice (average neuronal activity of intermediately active cells at week 2: WT 2.31 (CI 2.19–2.47) transients/min, APPPS1 2.59 (CI 2.49–2.69) transients/min, *p* < 10^−3^ Mann–Whitney-U test), rendering them more likely to transition into highly active cells. Thus, “hyperactivity” results from a gradual increase in activity of mainly intermediately active cells and not by a rapid, pronounced transition in activity of individual cells in awake APPPS1 mice.

### Effect of amyloid plaque proximity

**Fig. 5:**
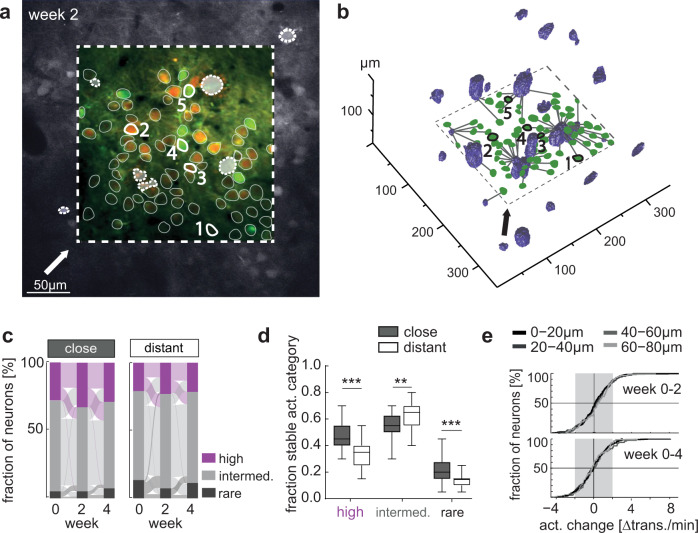
Impact of amyloid plaque proximity on the dynamics of neuronal activity. **a** Representative FOV (dashed rectangle) within a plane of a z-stack (shown is the mRuby2 channel in grey scale), used to measure the distances between plaques and neurons; GCaMP6s (green), mRuby2 (red), plaques (location of plaques is derived from the methoxy-XO4 channel and indicated by dashed lines). Selected ROIs are identical in **a**, **b**. The arrow indicates the viewing angle in **b**. **b** 3D reconstruction of the imaged area shown in **a**; plaques (blue) and the ROIs (green). Lines connecting ROIs and plaques represent the shortest distance between a neuron and the closest plaque border. **c** Relative proportions of highly, intermediately and rarely active neurons and their fractional change over time in APPPS1 mice for close (<=40 µm) and distant (>40 µm from plaque border) neurons. Relative proportions of activity categories did not differ and were stable over time (fraction highly active neurons: effect of group: *F*_1,56_ = 0.44, *p* = 0.51, effect of time: *F*_2,56_ = 0.13, *p* = 0.88, group-by-time interaction effect: *F*_2,56_ = 0.78, *p* = 0.46, fraction of intermediately active neurons: effect of group: *F*_1,56_ = 0.18, *p* = 0.68, effect of time: *F*_2,56_ = 0.5, *p* = 0.61, group-by-time interaction effect: *F*_2,56_ = 1.14, *p* = 0.33, fraction rarely active neurons: effect of group: *F*_1,56_ = 0.68, *p* = 0.41, effect of time: *F*_2,56_ = 2.62, *p* = 0.08, group-by-time interaction effect: *F*_2,56_ = 1.16, *p* = 0.32; two-way repeated measures ANOVA, close *n* = 15, distant *n* = 15 experiments (containing 326 neurons close, 326 neurons distant)). **d** Fraction of neurons in each activity category that remain within the same category over the whole imaging period (close (gray) and distant (white) neurons, highly active neurons *p* < 10^−4^, intermediately active neurons *p* = 0.005, rarely active neurons *p* < 10^−3^_,_ neurons pooled, data are median ± 95% CI). **e** Activity change distribution for neurons within different plaque-distance bins. The majority of neurons (>75%) changed their activity within a range of ±2 transients/min (gray area) (week 0–2: *F*_3,615_ = 0.47, *p* = 0.7, one way ANOVA; week 0–4: *F*_3,615_ = 0.97, *p* = 0.41, one way ANOVA, neurons pooled). **P < 0.01, ***P < 0.001.

Aβ plaques are a hallmark of AD pathology, and even though their pathogenic relevance is currently debated^29^, the local plaque environment is associated with a number of pathological features, such as dystrophic neurites, synapse instability and loss^16^, reactive microglia and astrocytes and hyperactive neurons^1,30–32^. We thus asked whether neurons close to plaques would change their activity more vigorously or frequently than neurons further away from plaques. To this end, we measured the distance of each neuron to the nearest plaque, stained by the dye Methoxy-XO 4 in 3D (Fig. 5a, b). In this analysis, only experiments, in which we could faithfully track the distance to the nearest plaque for all neurons at all imaging time points were included. Based on the median distance of all neurons to the nearest plaque (Supplementary Fig. 6a, week 0: 39.9 µm, week 2: 36.8 µm, week 4: 34.9 µm—plaque distance decreases as plaques grow over time^28,33^), we divided neurons into close (<=40 µm from the plaque border) and distant (>40 µm from plaque border) from the nearest plaque, respectively (of note, the effect is also true for other cut offs, such as 20, 60 or 80 µm). The fraction of highly or rarely active neurons did not differ significantly between close and distant neurons at the level of individual experiments (Supplementary Fig. 9a). Relative proportions of highly, intermediately, and rarely active neurons in APPPS1 mice were also stable for both close and distant neurons (Fig. 5c). However, neurons close to plaques that were either highly or rarely active, were more likely to maintain their activity level than those neurons further away from plaques (Fig. 5d, Supplementary Data 11). Intermediately active neurons, on the other hand, were more stable when they were located further away from plaques (Fig. 5d). When analyzing the change in neuronal activity for neuronal populations divided into plaque distance bins, we found that the majority of neurons preserve their activity, an effect that was independent of the plaque proximity (Fig. 5e, Supplementary Data 12). We also investigated whether new plaques^34^ were formed in close proximity to the neurons, we were recording from, to assess their impact on neuronal activity. However, in line with previous reports^33,35^ in our data set most of the newly formed plaques were very small and appeared very close to pre-existing ones or were too far away from the imaged focal plane to cause a major impact on the proximity of imaged neurons to the nearest amyloid plaque (see Supplementary Fig. 7). We thus could not address the question of how newly formed amyloid plaques would affect the activity of nearby neurons.

Taken together, our results indicate that over the course of four weeks single cell neuronal activity in awake APPPS1 mice varies only slightly, is as stable as in WT mice, and is independent of plaque proximity.

**Fig. 6:**
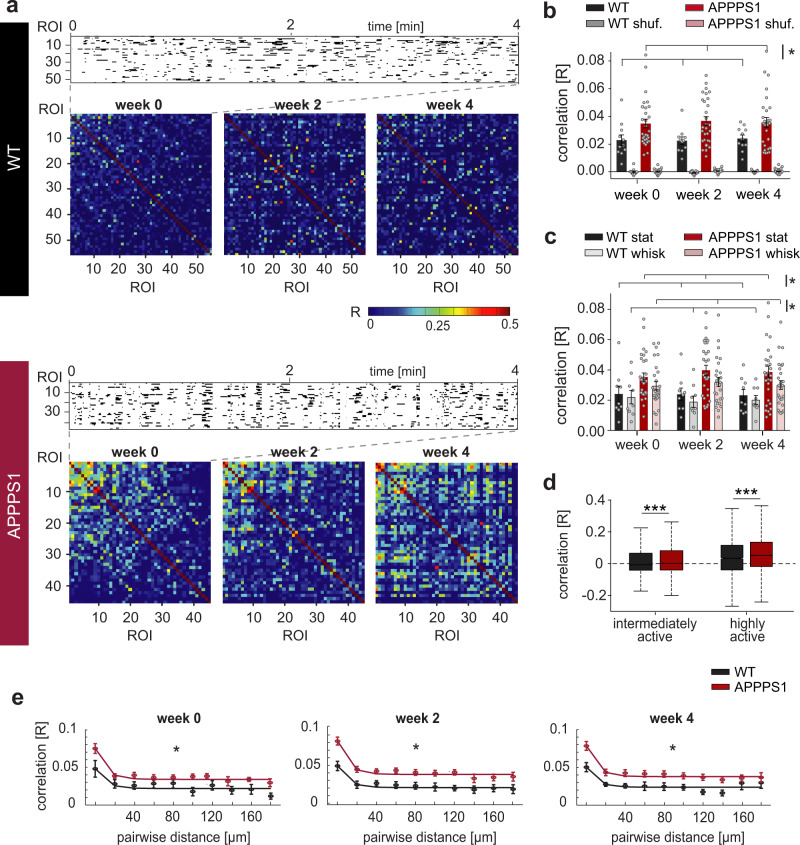
Increased pairwise activity correlation in APPPS1 transgenic mice. **a** Representative example of a raster plot depicting binarized neuronal activity (upper panel) in both a WT and an APPPS1 mouse. Pairwise correlations (Pearson’s correlation coefficient R) were computed and sorted at the first time point and displayed as color-coded correlogram for each time point (bottom of each panel) for the WT (upper panel) and the APPPS1 (lower panel) mouse. **b** Average neuronal correlation in individual experiments in WT (black) and APPPS1 (red) mice for actual experimental (bright color bars) and shuffled (pale bars) data. The Pearson’s correlation coefficient (*R*) was significantly increased in APPPS1 mice compared to WT mice (WT vs APPPS1: effect of group: *F*_1,72_ = 6.44, *p* = 0.015, time: *F*_2,72_ = 0.97, *p* = 0.38, group-by-time interaction: *F*_2,72_ = 0.57, *p* = 0.57; WT vs WT shuffled, group: *F*_1,72_ = 43.66, *p* < 10^−9^, time: *F*_2,72_ = 0.18, *p* = 0.83, group-by-time interaction: *F*_2,72_ = 0.08, *p* = 0.93; APPPS1 vs APPPS1 shuffled, group: *F*_1,72_ = 167.52, *p* < 10^−9^, time: *F*_2,72_ = 1.41, *p* = 0.25, group-by-time interaction: *F*_2,72_ = 1.19, *p* = 0.31, two-way repeated measures ANOVA, WT *n* = 9 (5 mice), APPPS1 *n* = 29 experiments (9 mice)). **c** Average pairwise correlations during stationary (stat) and whisking (whisk) epochs in WT and APPPS1 mice (WT stationary vs WT whisking: effect of group *F*_1,32_ = 0.46, *p* = 0.51, WT stat *n* = 9 and WT whisk *n* = 9 experiments (5 mice); APPPS1 stationary vs APPPS1 whisking: group: *F*_1,108_ = 3.77, *p* = 0.057, APPPS1 stat *n* = 29 and APPPS1 whisk *n* = 29 experiments (9 mice); WT stat vs APPPS1 stat: group: *F*_1,72_ = 6.06, *p* = 0.019, time: *F*_2,72_ = 1.08, *p* = 0.34, group-by-time interaction: *F*_2,72_ = 0.42, *p* = 0.66; WT whisking vs APPPS1 whisking: group: *F*_1,68_ = 5.21, *p* = 0.029; time: *F*_2,68_ = 0.16, *p* = 0.86, group-by-time interaction: *F*_2,68_ = 0.49, *p* = 0.61, all two-way repeated measures ANOVA). **d** Pairwise correlations during stationary epochs between highly active (*p* < 10^−16^, WT *n* = 3198 neuronal pairs, APPPS1 *n* = 37132 neuronal pairs) and intermediately active neurons (*p* < 10^−37^, Mann–Whitney-U test, WT n = 36970 neuronal pairs, APPPS1 n = 99759 neuronal pairs). **e** Neuronal correlation as a function of distance between neuronal pairs in WT (black) and APPPS1 (red) mice for three imaging time points (week 0: effect of group *F*_1,2_ = 21.4, *p* = 10^−5^, distance: *F*_2,9_ = 7.42, *p* = 10^−8^, week 2: group *F*_1,239_ = 51.12, *p* = 10^−11^, distance: *F*_9,239_ = 10.17, *p* = 10^−12^, week 4: group *F*_1,239_ = 32.76, *p* = 10^−7^, distance: *F*_9,239_ = 7.41, *p* = 10^−9^, two-way ANOVA, WT *n* = 7 experiments, APPPS1 *n* = 23 experiments, only experiments with pairwise distances covering the whole range of distance bins were considered). Solid line represents an exponential fit for visual guidance. At all imaging time points we found higher pairwise correlations in APPPS1 at all neuronal distances, (data in **b**, **c**, **d**, **e** is mean ± SEM). *P < 0.05).

In addition to alterations in neuronal activity, pathological changes could also influence the temporal correlation of activity between neurons. We thus investigated the synchrony of neurons recorded in each field of view (Fig. 6a). As fluctuations within the neuropil are often highly correlated (Supplementary Fig. 8) and can potentially contaminate the signal sampled from the soma, we computed the Pearson’s correlation coefficient (*R*) of binarized, neuropil compensated fluorescent traces (see Methods and Supplementary Fig. 8). Independent from the neuropil compensation factor applied, the pairwise activity correlation was significantly increased in APPPS1 mice compared to WT mice across all time points (Fig. 6b, Supplementary Data 13, Supplementary Fig. 8g). As different internal brain states associated with quiet wakefulness or whisking can strongly affect the synchrony of neuronal activity^36^, we separately probed pairwise correlations during stationary and whisking epochs. There was an overall trend towards lower pairwise correlations during whisking, but this effect was not significant in WT mice (Fig. 6c) and was only marginally significant in APPPS1 mice (Fig. 6c, Supplementary Data 14). Nevertheless, the genotype difference was evident, with APPPS1 mice displaying higher levels of neuronal synchrony during both stationary and whisking epochs (Fig. 6c). In addition to cortical states, also overall neuronal activity levels can influence pairwise correlations. To this end, we computed pairwise neuronal activity correlations separately for highly active and intermediately active cells in WT and APPPS1 mice. We again observed a higher synchrony level in APPPS1 mice for highly active and for intermediately active cells (Fig. 6d).

Finally, we asked whether neuronal ensembles with a high degree of synchrony are also located in close proximity to each other, by testing whether the pairwise correlation was dependent on the physical distance of the neurons (Fig. 6e). Consistent with previous work^37^, we observed a higher correlation between neighboring neurons (within 20 µm^37^). Notably, pairwise correlations in APPPS1 were higher than in WT mice at all distances (Fig. 6e). Collectively, our results demonstrate that neurons in APPPS1 transgenic mice exhibit an enhanced level of correlated activity.

## Discussion

By monitoring the same individual neurons over four weeks in awake APPPS1 transgenic mice and their non-transgenic littermates, we demonstrate that (a) a larger fraction of neurons was highly active in awake APPPS1 compared to WT mice, (b) the activity levels of individual neurons largely persisted across the investigational period, (c) a fraction of intermediately active cells in APPPS1 mice slowly increased their activity over time, resulting in the development of highly active cells (Supplementary Fig. 9b), and (d) altered neuronal activity is associated with a higher pairwise correlation in awake APPPS1 mice during both quiet and active (whisking) epochs.

Aberrant activity levels have been documented in mouse models of AD and human patients^1,2,4,17^ and were shown to impair neural network function^5,23^. Network alterations are in turn believed to underlie the cognitive deficits typical of AD. Restoring single cell activity levels and neural circuit function thus represents a promising therapeutic target to mitigate the cognitive decline in AD. However, one of the main features proposed to drive network dysfunction, namely hyperactive neurons, have so far been mainly studied in anesthetized mice, employing acute preparations, thus leaving open whether they exist during wakefulness and, importantly, what the fate of aberrantly active cells might be. Previous in vivo imaging studies used low dose isoflurane as an anesthetic and the synthetic calcium indicator Oregon-Green Bapta-1 AM (OGB1) to investigate neuronal and astrocytic calcium responses in mouse models of cerebral amyloidosis, such as APP23 x PS45^1,5,17^, APPswe x PS1 dE9^31,32^, APP Tg2576 and PDAPP mice^6^. Our study goes beyond these reports, as we investigate the activity of the same individual neurons *repeatedly* over a period of four weeks and in *awake* mice. Anesthesia is known to alter neuronal function in a concentration-dependent manner. While high doses mainly dampen neuronal activity and functional connectivity entirely^37,38^, lower doses can cause unexpected alterations. As such, volatile anesthesia have been shown to strongly reduce particularly the inhibitory drive during sensory processing^39^, to increase network synchrony and to alter neuronal response properties^37,38^. We here thus recorded neuronal activity during quiet wakefulness in mice trained to sit still in a restrainer. Also under these conditions, we found a larger fraction of neurons in APPPS1 mice displaying high activity levels, already at young ages, when little plaque load is present. This finding is in agreement with a previous study, which reported activity alterations in cortex as early as 4.5 months of age in a similar APP/PS1 transgenic mouse model^5^. Importantly, by following individual neurons over time, we now also demonstrate that hyperactivity is a stable feature, present over at least four weeks during the early phase of cerebral amyloidosis in APPPS1 mice. We also observed some fluctuations in the average neuronal activity both in WT and in APPPS1 mice, which could cause a shift in activity category assignment. These fluctuations are expected and can, even under physiological conditions, be as large as an order of magnitude^40^. Despite these fluctuations the fraction of highly active neurons was consistent over the 4-week investigation period and the reoccurrence rate for highly active cells was around 60% over 2 weeks, indicating an overall stability of aberrant activity levels. Notably, we found a slight increase in absolute neuronal activity, particularly for intermediately active cells, which ties in with a larger fraction of intermediate cells turning highly active in APPPS1 mice. If the slight increase in average activity (~0.5 transients/min within 2–4 weeks) of intermediately active neurons persisted, it could well serve as a source for future highly active cells.

Our data also suggest that almost all cells, we recorded from, are in fact active cells, since the reoccurrence rate of rarely active cells in both WT and APPPS1 was close to zero after 4 weeks. The fraction of rarely active cells was also low and did not significantly differ between WT and APPPS1, indicating that at this early stage of the amyloidosis silent cells do not yet exist during wakefulness. Moreover, we did not find any compelling evidence for highly active cells turning into rarely active neurons over the four-week imaging period (only 1 out of 471 in WT and 4 out of 1514 total neurons in APPPS1 mice turned from highly active into rarely active cells over 4 weeks). Thus, our data argue for a temporal sequence, in which during early stages of the disease neuronal activity is elevated and possibly only at later stages some neurons turn rarely active or silent. Overall, the here proposed slow process of aberrant activity development would fit well with progressive network dysfunction based on E/I imbalance^23^. This notion is further corroborated by the observed increased correlation of neuronal activity in APPPS1 mice, as enhanced neuronal synchrony argues for circuit-level defects (such as increased synaptic inputs or compromised inhibition). Previous work has reported epileptic discharges in APP transgenic mice, which the authors linked to dysfunctional parvalbumin (PV) positive interneurons^4^. However, the level of synchrony we observed in awake mice, is unlikely to be caused by epileptiform discharges, as in that case one would expect even higher pairwise correlations (‘hypersynchrony’)^41^. It might instead result from aberrantly synchronized excitatory inputs, due to structural remodeling that has been shown to accompany for instance amyloid plaque pathology^16,42^. In line with this idea are reports of increased local connectivity and synchronization in AD, while inter-regional connectivity was strongly decreased^43^. Interestingly, similar alterations in cortical neuronal excitability were also recently described in other mouse models of neurodegenerative diseases (NDs), such as Huntington’s disease^44^, potentially indicating that impaired E/I balance constitutes a general feature in NDs.

Earlier work has suggested that highly active cells are spatially linked to plaque proximity^1^. This finding, however, remains controversial, as it could not be replicated in other studies^5,23^. The immediate plaque proximity is characterized by a number of structural and functional aberrations, such as dendritic spine instability and loss^16,45^, neuritic dystrophies, neurite breakage^46^, increased calcium concentration within dendrites^32^, and glia activation^31,47^. Amyloid plaques have been shown to contain high concentrations of soluble Aβ^48^, which in turn might trigger neuronal hyperactivity^17^. We thus investigated neuronal activity levels and their dynamics separately for neurons close to plaques (0–40 µm away from the closest plaque border) and those further away. Although there was a trend towards a higher fraction of highly active and a lower fraction of rarely active neurons close to plaques, this effect was not significant. As cortical neurons are strongly connected laterally^49^, it is conceivable that the local impact of amyloid plaques and/or Aβ on neuronal activity is less restricted and rather propagates through strong lateral connections. Notably, we however found differences in the stability of the neuronal activity levels. Close to plaques neurons were more likely to remain either highly or rarely active, while distant from plaques more cells would remain intermediately active, indicating a ‘solidification’ of aberrant activity levels close to plaques, while neurons further away might still be in a transition phase.

One of the important questions our findings raise, is why homeostatic mechanisms seem to fail in AD. Irrespective of the kind of perturbation, neurons, and neural circuits are typically bound to maintain a given ‘pre-determined’ average firing rate through processes collectively termed integrated homeostatic network^50^, which are apparently compromised in AD. Possible mechanisms might involve a failure of involved sensors, modulators, or feedback mechanisms. Altered calcium levels in AD could play a cardinal role in this process as CaMKIV, a kinase sensing changes in Ca^2+^ influx into a cell, has been shown to be of high relevance in regulating intrinsic excitability and synaptic scaling upon perturbations under physiological conditions^51^. These recently emerging concepts warrant further scrutiny, as they also pinpoint towards downstream circuit alterations that might eventually occur independently of Aβ levels and would consequently not be amenable to Aβ targeting therapeutic strategies. This notion agrees with our observation that activity levels are rather stable, despite a severe 9-fold increase in soluble/insoluble Aβ during the early stages of the disease.

Taken together, our data provide evidence that aberrant neuronal activity does exist during wakefulness, is a durable, single cell feature present over at least four weeks in APPPS1 mice and emerges slowly by a gradual increase of intermediary active cells.

## Methods

### Animals

All procedures were carried out in accordance with an animal protocol approved by the Ludwig-Maximilians-University Munich and the government of Upper Bavaria (ref number AZ: 55.2-1-54-2532-163-13).

As an AD transgenic model, the double transgenic mouse line APPPS1^27^ on a C57BL/6 J genetic background was used. These mice co-express the mutant amyloid precursor protein (APP, Swedish double-mutation KM670/671NL) and mutant presenilin 1 (PS1, L166P) under the control of the neuron-specific Thy-1 promoter (referred to as APPPS1). The line is hemizygous for both transgenes. Non-transgenic (wild type) littermates were used as controls (referred to as WT). Both sexes were used in the study. In total 9 APPPS1 (29 field of views (FOVs), 1514 neurons, 4 females, 5 males) and 5 WT mice (9 FOVs, 471 neurons, 3 females, 2 males) were included in the in vivo experiments and additional 10 APPPS1 mice (5 males at the age of 4 months and 5 males at the age of 5 months) were used for biochemical analysis.

Before the cranial window implantation surgery mice were housed in groups of three to six individuals in standard cages, with standard bedding and additional nesting material. After the surgery, mice were singly housed in standard cages. Food and water were provided ad libitum. Mice were kept under a 14/10-hr light/dark cycle.

### Biochemical characterization of mouse brain tissue

Mouse hemispheres from 4 and 5 months old APPPS1 animals were dissected and snap frozen in liquid nitrogen. Frozen hemispheres were processed similarly as previously described^52^. Briefly, hemispheres were homogenized in STET lysis buffer (150 mM NaCl, 50 mM Tris pH 7.5, 1% Triton-X) supplemented with protease and phosphatase inhibitor cocktail (Roche) and lysed for 30 min by shaking at 4 °C. Lysates were then centrifuged at 17,000 *g* for 30 min at 4 °C and supernatants were collected (STET fraction, capturing soluble Aβ). Pellets were further resuspended in 70% formic acid, sonicated for 5 min and centrifuged for 1 h at 100,000 *g* and 4 °C. Supernatants were neutralized 1:20 in 1 M Tris-HCl, pH 9.5 (FA fraction, capturing insoluble Aβ). Western blot analysis and Aβ detection were performed as described, using the 2D8 antibody^53^. Antibody against β-actin was used as a loading control (1:1000, A5316, Merck).

### Surgery

For in vivo imaging, a chronic cranial window was implanted as described previously^28^. Mice underwent surgery at the age of 3 months. Briefly, mice were anesthetized by an intraperitoneal injection of Ketamine/Xylazine (14 mg/kg body weight; WDT/Bayer Health Care). Additionally, Dexamethasone (6 mg/kg body weight; Sigma) was intraperitoneally administered immediately before surgery. Firstly, a round craniotomy of 3 mm in diameter was made above the right hemisphere frontal to bregma (coordinates of the center of the craniotomy: 1.5 mm anterior, 1.75 mm lateral to bregma) using a dental drill (Schick-Technikmaster C1; Pluraden; Offenbach, Germany). Then the virus injection was performed within the center of the craniotomy. The virus AAV2.1.hSyn1.mRuby2.GSG.P2A.GCaMP6s.WPRE.SV4^25^; (Cat.No 50942-AAV1 Penn Vector Core) was injected at 1:50 dilution of the original stock (final virus titer 0.33 × 10^13^ GC ml^−1^) at a volume of 300 nl each in 3–5 injections at a depth of 0.8 mm, at a speed of 33 nl/min using the NANOLITER 2010 Injector with Micro4 Controller (World Precision Instruments).The injection site was selected such that no big blood vessels would be damaged by the injection. Immediately after all the 3–5 injections were done the craniotomy was covered with round coverslip (3 mm, 0.16–0.19 mm thickness, World Precision Instruments). The coverslip was sealed using dental acrylic (Cyano-Veneer fast; Schein). A custom-made small metal bar was attached with dental cement next to the coverslip to allow for a stable head-fixation during training and awake imaging sessions. After surgery, mice received subcutaneous doses of the analgesic Carprofen (7.5 mg/kg; Pfizer) and the antibiotic Cefotaxime (5 mg/kg; Pharmore).

### Longitudinal awake in vivo two-photon imaging

Imaging was performed in awake, head-fixed mice, sitting in a restrainer. Mice were trained to accommodate to the head-fixation for 14–21 days prior to imaging. The training consisted of daily sessions, of which the first 3 days mice were simply handled by the researcher, exposed to the setup and allowed to freely explore the restrainer and the head-holder. On the subsequent two days mice were only briefly head-fixed (10–30 s), after which the duration of head-fixation was gradually increased for up to one hour at the end of training. The number of training days was adjusted individually, until mice showed no signs of distress and remained still during the head-fixation period. Weekly imaging sessions started four weeks after the surgery to allow mice to recover and accommodate to the setup and the cranial windows to become stable. If the mouse was not getting habituated to the setup and showed signs of distress during fixation after the training period, it was removed from the experimental group.

Around 5–15 h before each imaging session, Methoxy-X04 (Xcessbio, San Diego, CA, USA, 3.3% vol of 10 mg/ml stock solution in DMSO (light shielded), 6.66% vol Cremophore EL (Sigma Aldrich) in 90% vol PBS), was intraperitoneally injected at a concentration of 3.33 mg/kg body weight to stain amyloid plaques in vivo^54^. Before each imaging session, mice were head-fixed and placed under the microscope for 5 min to habituate. Imaging was performed in the dark without any additional stimuli. In each mouse, two to six regions at depths of 120–200 µm below the pial surface (layer 2/3) of the frontal cortex were imaged. All imaging regions were located within a circular 2 mm-diameter area centered at 1.5 mm anterior from Bregma and 1.75 mm lateral from the midline, thus covering M1 and M2 cortical areas. In vivo time-lapse imaging stacks were acquired at a frame rate of 10 Hz using the LaVision Trim Scope microscope equipped with 2 tunable Ti:sapphire two-photon lasers (Coherent Chameleon and Mai Tai Spectra Physics). The setup was controlled using LaVision Imspector software (LaVision Biotech, Germany). The Chameleon laser was tuned to 940 nm, which enabled simultaneous excitation of mRuby2 and GCaMP6s. A 25x, NA 1.05 water-immersion objective (Olympus) was used. For the first cohort the imaging dimensions were 173 × 173 pixels; corresponding to 151 × 151 μm, for the second cohort—223 × 223 pixels and 220 × 220 μm. At each session the same cells for each area of interest were imaged over 5000 frames (8.3 min duration).

For the analysis of plaque distances z-stacks capturing the surrounding tissue of the imaged area were acquired, with each stack covering 250–350 µm in depth (520 × 520 pixels; *x*,*y* dimensions: 350 μm, *z* increments 0.5 µm). The simultaneous excitation of Methoxy-X04 was achieved by the Mai Tai laser tuned to 750 nm. During the z-stack acquisition mice were anesthetized by 0.5 vol % isoflurane after the awake imaging session. At all times laser power was kept below 80 mW measured at the back-focal plane of the objective. Emitted fluorescence light was split at 495 nm and 560 nm, to separate the emitted light from Methoxy-XO4, GCaMP6s and mRuby2 and detected by photomultiplier tubes. During head-fixation, mice typically showed long episodes of quiet wakefulness (quiet phase) interrupted by brief episodes of whisking or grooming (active phase). We recorded the behaviour of the mouse by a webcam, controlled by the LaVision Imspector software, which allowed for a synchronisation of the two-photon and the behavioural recordings. Neuronal activity associated with whisking was analysed separately from stationary epochs. Whisking events varied in duration (1 s on average in our data) and are partly too short to be precisely temporally aligned with the calcium events we obtain using GCaMP6. To this end, whisking events lasting at least 330 ms (that is 3 frame times) where identified and neuronal activity was measured within a window of 1 s up to 2 s upon whisking offset. Stationary neuronal activity was assessed during non-whisking/grooming epochs.

### Immunohistochemical identification of inhibitory neurons

The PFA-fixed brains were cut on a vibratome (Leica VT 1000 S) into 50 µm thick coronal sections. Immunohistochemistry was performed on free-floating sections. Sections were incubated overnight in 2% Triton X-100 in PBS at room temperature for permeabilization of the tissue, then blocked for 2 h at room temperature with 3% I-Block™ Protein-Based Blocking Reagent (Thermo Fisher Scientific) containing 0.2% Triton X-100 in PBS, before they were incubated with the primary antibodies (mouse anti-GAD67 in 1:500 dilution (Millipore, catalogue number MAB5406B)) overnight. Incubation with the secondary antibody (goat anti-mouse Alexa Fluor 647 1:500, Life Technologies, catalogue number A21236) was conducted for 2 days at room temperature. Confocal stacks (Zeiss LSM 780) were acquired first at a low magnification (10x objective) to allow for identification of virus-transfected regions. Confocal stacks of re-identified imaging spots were then acquired with a 40x objective. Analysis of images was performed using ZEN software (Zeiss) in raw z-stacks by manually scrolling through respective frames and marking mRuby2- and GAD67-positive neurons.

### Image data processing and analysis

Collected images were processed and analysed using custom written routines in MATLAB (The MathWorks, Inc.) and ImageJ (http://rsb.info.nih.gov/ij/). Image pre-processing for calcium imaging data was done by custom-made script^37^. In brief, in vivo two-photon recordings were corrected for slight brain displacement artefacts in the *x*–*y* plane by realigning the images. Recordings with clear movement artefacts along the *z*-axis were excluded from further analysis. Regions of interest (ROIs) were outlined semi-automatically based on a maximum projection of all frames for all neurons with the help of custom-made GUI for each repetition block separately (see https://github.com/JorritMontijn/Preprocessing_Toolbox). Indicator-filled cells - seen as bright cells in the GCaMP6 channel without the nucleus being spared at all times of the recordings - were excluded from analyses (5% of all neurons in WT and 6.1% in APPPS1 mice) as their calcium kinetics differ from a pure cytosolic expression^26^. The fluorescent intensity of all pixels contained within a given ROI was averaged for each frame for both channels. To correct for contamination by the neuropil, a region surrounding the selected ROI was selected and the average fluorescent intensity within that area was calculated for each frame. The corrected ROI signal was computed based on the equation^23,26^:

$${{{{{{\rm{F}}}}}}}_{{{{{{\rm{ROI}}}}}}\_{{{{{\rm{comp}}}}}}}={{{{{{\rm{F}}}}}}}_{{{{{{\rm{ROI}}}}}}}+0{{{{{\rm{.7x}}}}}}({{{{{\rm{median}}}}}}({{{{{{\rm{F}}}}}}}_{{{{{{\rm{neuropil}}}}}}})-{{{{{{\rm{F}}}}}}}_{{{{{{\rm{neuropil}}}}}}})\left.\right)$$

F_ROI_comp_ represents the actual signal within the selected ROI after compensating for neuropil contamination, while F_ROI_ reflects the signal within the initially selected ROI. F_neuropil_ corresponds to the signal within the surrounding neuropil. Traces were next low pass filtered at 5 Hz and slow fluctuations removed by subtracting the 8th percentile within a window of ±50 s. In order to estimate F0, we subtracted the 8th percentile in a very short window of 1 s and used the median of all values below the 60th percentile of this ‘noise band’ as F0. This procedure allowed for reasonable F0 detection for both highly and rarely active cells. To classify a neuron as active, it had to display at least one prominent transient exceeding the threshold of F0 plus 3x the standard deviation of the noise band for more than 9 frames (equaling 1 s). Transients were identified on traces smoothed over 5 frames and had a minimum distance of 15 frames (1.5 s) and a minimum height of 3x standard deviation of the noise band. The classification into activity groups is based on the frequency of transients: rarely active < 0.25 transients/min; intermediately active 0.25–4 transients/min; highly active >4 transients/min^1^. The correlation (Pearson’s *R*) of the activity between ROIs was based on binary traces. To this end the traces were smoothed across 20 frames (2 s) and binarized at a threshold of 2x standard deviation. The distance between cells reflects the distance of the ROIs’ centroids.

### Plaque distance analysis

The analysis of the amyloid plaque distances was carried out in MATLAB (MathWorks) using custom written routines. The position and size of the ROIs were projected into the 3D rendered overview stack. The two channels carrying either the mRuby2 or the Methoxy-XO4 signal were background subtracted, and subsequently the GCaMP6s channel was subtracted from the Methoxy-XO4 channel in order to remove slight bleed through. Each frame of the Methoxy channel was median filtered and binarized using the background plus 3x standard deviation as threshold. Plaque distance is the 3D Euclidean distance between the centroid of the respective ROI and the nearest Methoxy positive voxel. All overview stacks were visually checked for correct plaque detection and 3D rendered stacks were inspected for accurate neuron—plaque assignment (ruling out accidental pairing with voxels carrying signal stemming from the dura (due to generation of second harmonics)). The distances resulting from an incorrect assignment of plaque voxels were exchanged by manually measured distances. Manual measurement was done between the centroid of the respective ROI and the nearest Methoxy positive voxel using the manual measurement tool in ImageJ (http://rsb.info.nih.gov/ij/), taking into account the Pythagorean Theorem in case the nearest plaque was not in the same plane as the ROI.

### Statistical analysis

If not stated otherwise in the text, neuronal activity and fractions of different activity categories and their changes over time were compared using a two-way repeated measure ANOVA. Distributions of activity changes were compared using a Kolmogorov–Smirnov (KS) test. Neuronal correlation was investigated by comparing the average correlation of each experiment (derived from all pairwise correlations within a field of view) for each genotype across all time points using two-way repeated measure ANOVA. *P*-values are reported as follows: *P < 0.05, **P < 0.01, and ***P < 0.001.

### Reporting summary

Further information on research design is available in the Nature Research Reporting Summary linked to this article.

## Supplementary information

Supplementary Information

Description of Additional Supplementary Files

Supplementary Data 1

Supplementary Data 2

Supplementary Data 3

Supplementary Data 4

Supplementary Data 5

Supplementary Data 6

Supplementary Data 7

Supplementary Data 8

Supplementary Data 9

Supplementary Data 10

Supplementary Data 11

Supplementary Data 12

Supplementary Data 13

Supplementary Data 14

Reporting Summary

## Data Availability

All data needed to evaluate the conclusions in the paper are present in the paper and/or the Supplementary Information. Additional data is available from lead contact upon reasonable request.
