# Supplementary material for: Long-term dynamics of aberrant neuronal activity in awake Alzheimer’s disease transgenic mice
Source: Commun Biol. 2021 Dec 7;4:1368. doi: 10.1038/s42003-021-02884-7 (PMC8651654; doi:10.1038/s42003-021-02884-7)
Supplement: Supplementary file 1 — Supplementary Information [file 42003_2021_2884_MOESM1_ESM.pdf]

Supplementary Information

Long-term dynamics of aberrant neuronal activity in awake Alzheimer's disease transgenic mice

V. Korzhova [1,2], P. Marinković [1,2], J. Rudan Njavro[1], P. M. Goltstein[5], F. Sun[1,2], S. Tahirovic[1], J. Herms[1,2,3] and S. Liebscher [3,4,6,*]

[1]German Center for Neurodegenerative Diseases (DZNE), 81377 Munich, Germany
[2]Center for Neuropathology and Prion Research, Ludwig-Maximilians University Munich, 81377 Munich, Germany
[3]Munich Cluster for Systems Neurology (SyNergy), 81377 Munich, Germany
[4]Institute of Clinical Neuroimmunology, Klinikum der Universität München, Ludwig-Maximilians University, 82152 Martinsried, Germany
[5]Max Planck Institute of Neurobiology, 82152 Martinsried, Germany
[6]Biomedical Center, Medical Faculty, Ludwig-Maximilians University Munich, 82152 Martinsried, Germany

* correspondence : sabine.liebscher@med.uni-muenchen.de

Content :

Supplementary Fig. 1. Example of *in vivo* imaging data.

Supplementary Fig. 2. Recorded neurons are mainly excitatory

Supplementary Fig. 3. Comparison of Aβ load in 4 and 5 month old APPPS1 animals

Supplementary Fig. 4. Activity of individual neurons over time

Supplementary Fig. 5. Loss and gain within activity categories

Supplementary Fig. 6. Distribution of plaque distances

Supplementary Fig. 7. Novel amyloid plaques form close to existing ones.

Supplementary Fig. 8. Impact of neuropil compensation on pairwise activity correlation

Supplementary Fig. 9. Fractions of rarely and highly active neurons close and distant from amyloid plaques

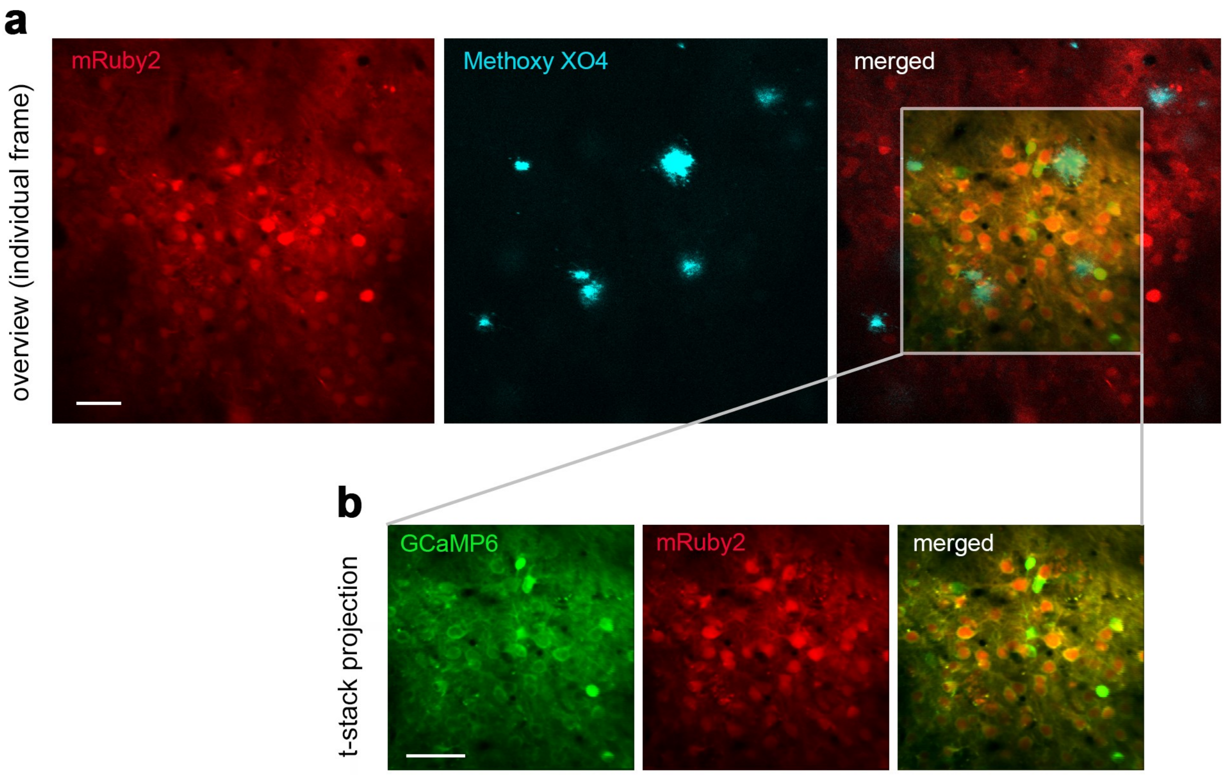

Supplementary Fig. 1. Representative example of in vivo imaging recordings.
(a) Individual frame of an overview z-stack, capturing the mRuby2 and the Methoxy XO4 signal. **(b)** Average projection of a t-stack, consisting of 6000 frames, capturing the GCaMP6s and the mRuby2 signal. Position of the t-stack is superimposed into the frame of the overview stack in (a). Scale bar in (a,b) 50um

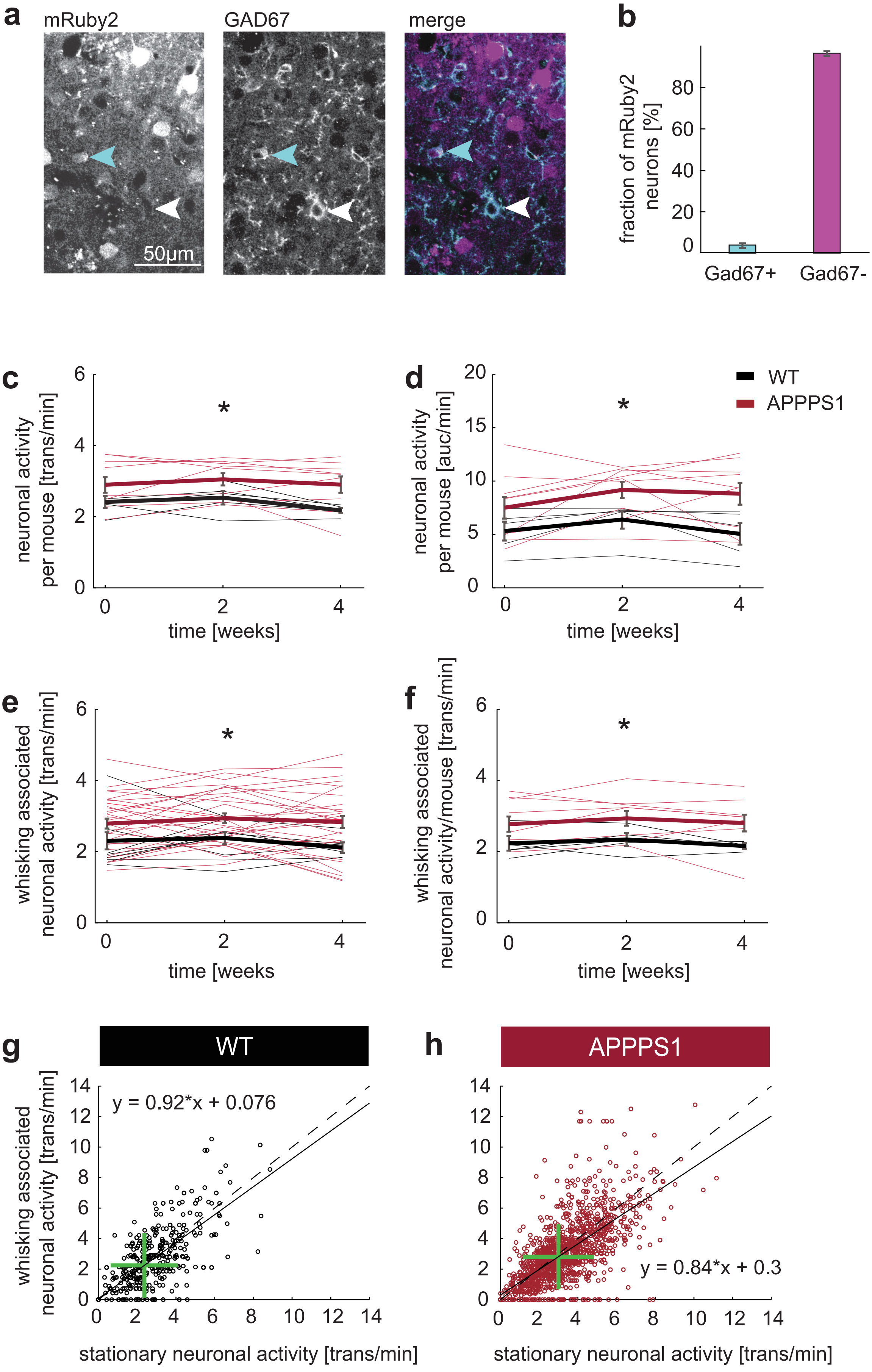

Supplementary Fig. 2. Recorded neurons are mainly excitatory.

(a) Immunohistochemical analysis of GCaMP6s-mRuby2-expressing neurons. Left, middle and right images represent confocal images of mRuby2, Gad67 immunohistochemical staining and merged image, respectively. White arrowhead marks a Gad67 positive neuron, cyan arrowhead points to an mRuby2 positive, Gad67 positive neuron. **(b)** Quantification of the relative proportion of Gad67 positive, mRuby2 expressing neurons. 3.57% of the mRuby2 expressing neurons (which co-express GCaMP6s) were positive for Gad67 (760 mRuby2-positive neurons, 3 mice, data are mean +/- SD). **(c)** Average neuronal activity during stationary epochs measured as transients per minute per mouse ($p < 0.05$ for all time points, non-parametric bootstrap method to compute difference in mean). **(d)** Average neuronal activity during stationary epochs measured as AUC per mouse (WT vs APPPS1: effect of group $F_{1,24} = 81.68$, $p = 0.046$, effect of time $F_{1,24} = 7.43$, $p = 0.09$, group-by-time interaction effect $F_{1,24} = 2.02$, $p = 0.49$). **(e)** Average neuronal activity associated with whisking per experiment (WT vs APPPS1: effect of group $F_{1,72} = 4.95$, $p = 0.032$, effect of time $F_{1,72} = 1.18$, $p = 0.31$, group-by-time interaction effect $F_{1,72} = 0.48$, $p = 0.62$, two-way repeated measures ANOVA). **(f)** Average whisking-associated neuronal activity per mouse ($p < 0.05$ for all time points, non-parametric bootstrap method to compute difference in mean). **(g)** Correlation between stationary and whisking related neuronal activity of individual neurons in WT mice and in **(h)** APPPS1 mice at week 0. The linear regression is plotted as black line (equation of the linear fit given in G, H, dashed line – unity line). The mean +/- SD is denoted as green cross in (G,H). Data are WT n = 9, APPPS1 n = 29 experiments (E) and WT n = 5, APPPS1 n = 9 mice (C, D,F).

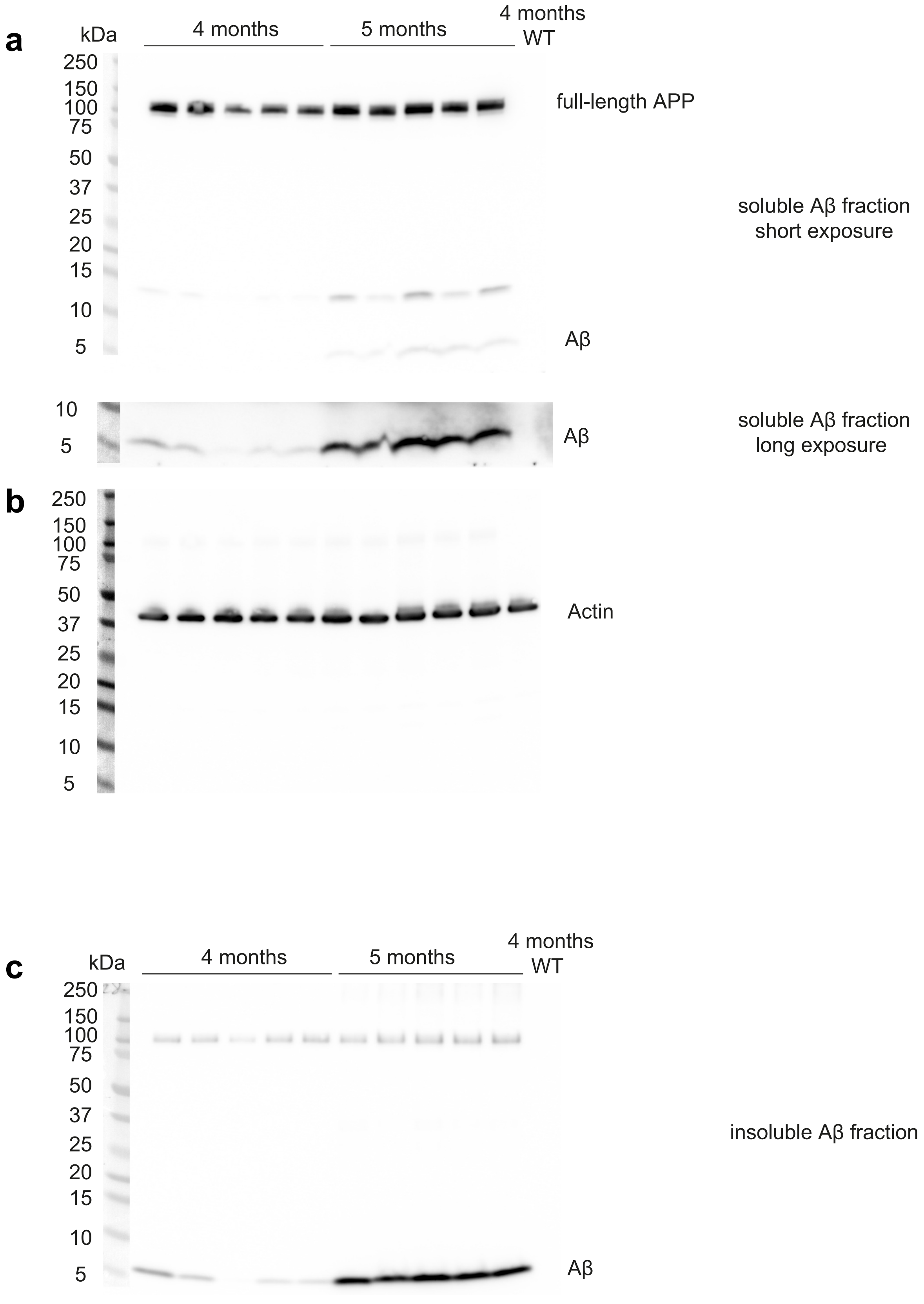

Supplementary Fig. 3. Comparison of Aβ load in 4 and 5 months old APPPS1 animals.
(a) Western blot analysis of the soluble Aβ fraction (n = 5 mice). Full blots are shown. A WT animal is used as a control for the human APP transgene and **(b)** actin is used as a loading control. Human Aβ was detected using the 2D8 antibody. A part of the digital version of the image containg the marker was added on the exposed image. **(c)** Western blot analysis of the insoluble Aβ fraction of the same samples shown in a,b.

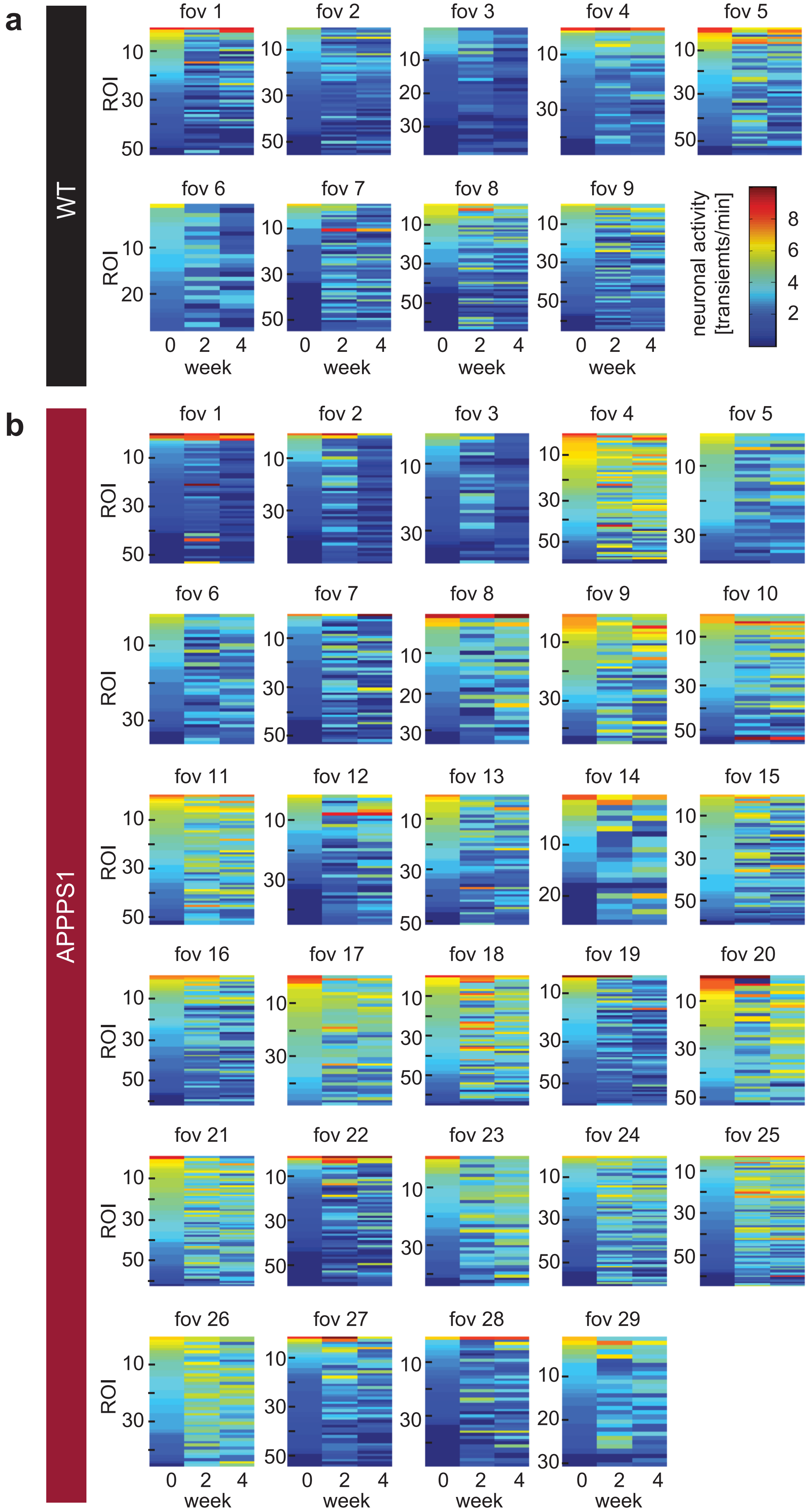

Supplementary Fig. 4. Activity of individual neurons over time. (a-b) Heat maps depicting color-coded activity of individual ROIs (each row represents a single ROI) over time, sorted at the first time point and the order is maintained for all imaging time points for each FOV in WT (a) and APPPS1 mice (b)

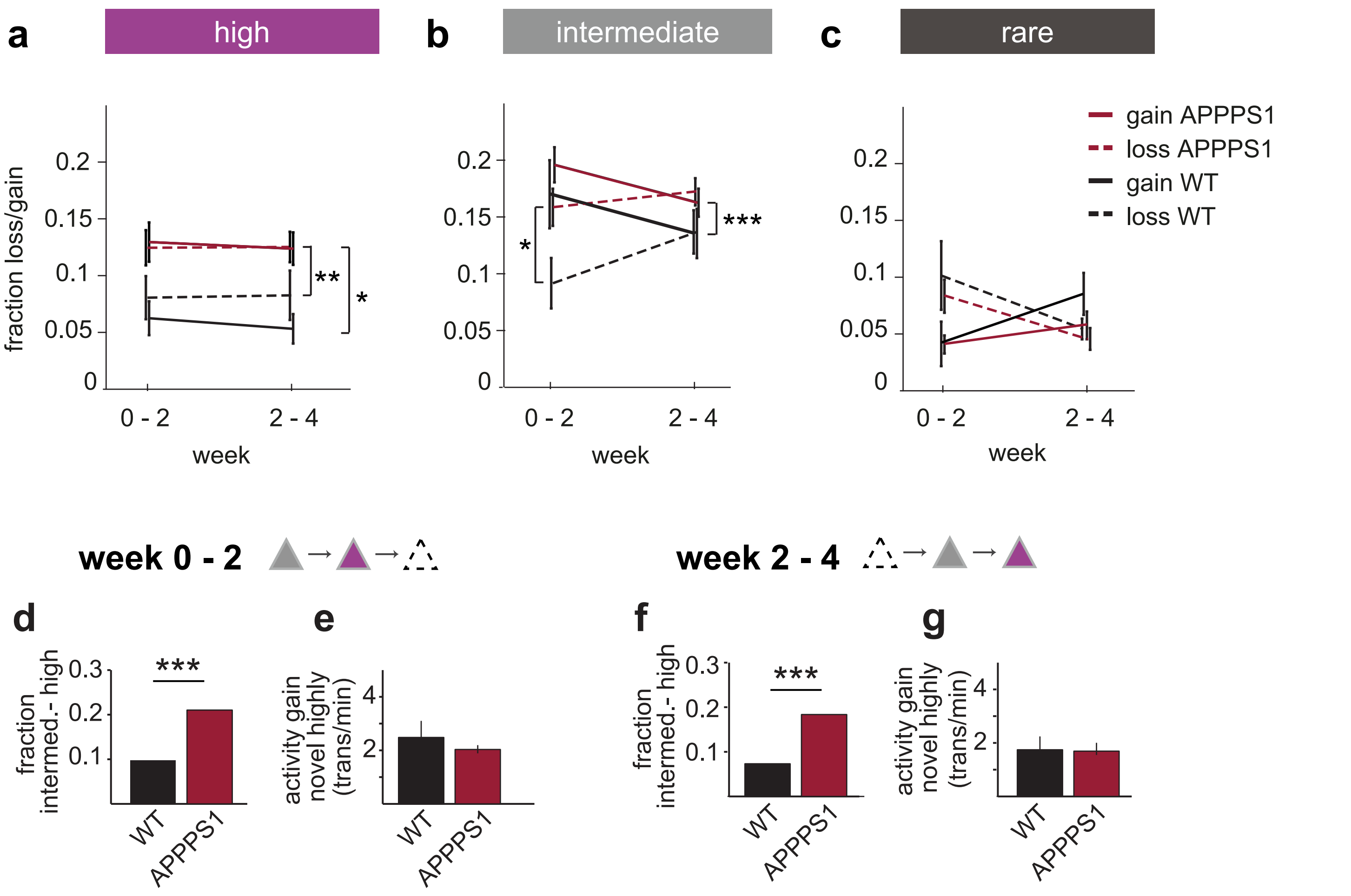

Supplementary Fig. 5. Loss and gain within activity categories. (a) Fraction of gained (solid lines) and lost (dashed lines) highly active neurons in WT (black) and APPPS1 (red) mice (fraction lost highly active cells WT vs APPPS1: effect of group $F_{1,38}$ = 4.65, p = 0.037, effect of time $F_{1,38}$ = 0, p = 0.95, group-by-time interaction effect $F_{1,38}$ = 0, p = 0.97; fraction gained highly active cells WT vs APPPS1: effect of group $F_{1,38}$ = 9.77, p = 0.003, effect of time $F_{1,38}$ = 0.22, p = 0.64, group-by-time interaction effect $F_{1,38}$ = 0.01, p = 0.91; gain vs loss in WT: effect of group $F_{1,20}$ = 1.76, p = 0.19, effect of time $F_{1,20}$ = 0.04, p = 0.83, group-by-time interaction effect $F_{1,20}$ = 0.11, p = 0.74; gain vs loss in APPPS1: effect of group $F_{1,56}$ = 0.01, p = 0.92, effect of time $F_{1,56}$ = 0.04, p = 0.85, group-by-time interaction effect $F_{1,56}$ = 0.06, p = 0.81). **(b)** Fraction of gained (solid lines) and lost (dashed lines) intermediately active neurons in WT and APPPS1 mice (fraction lost intermediately active cells WT vs APPPS1: effect of group $F_{1,38}$ = 6.37, p = 0.016, effect of time $F_{1,38}$ = 2.1, p = 0.16, group-by-time interaction effect $F_{1,38}$ = 0.83, p = 0.37; fraction gained intermediately active cells WT vs APPPS1: effect of group $F_{1,38}$ = 15.44, p = 0.0003, effect of time $F_{1,38}$ = 9.45, p = 0.004, group-by-time interaction effect $F_{1,38}$ = 4.13, p = 0.05; gain vs loss in WT: effect of group $F_{1,20}$ = 2.08, p = 0.16, effect of time $F_{1,20}$ = 0.07, p = 0.79, group-by-time interaction effect $F_{1,20}$ = 3.93, p = 0.06; gain vs loss in APPPS1: effect of group $F_{1,56}$ = 1.1, p = 0.3, effect of time $F_{1,56}$ = 0.43, p = 0.51, group-by-time interaction effect $F_{1,56}$ = 2.45, p = 0.12). **(c)** Fraction of gained (solid lines) and lost (dashed lines) rarely active neurons in WT and APPPS1 mice (fraction lost rarely cells WT vs APPPS1: effect of group $F_{1,38}$ = 0.51, p = 0.48, effect of time $F_{1,38}$ = 8.84, p = 0.005, group-by-time interaction effect $F_{1,38}$ = 0.1, p = 0.76; fraction gained rarely active cells WT vs APPPS1: effect of group $F_{1,38}$ = 0.69, p = 0.41, effect of time $F_{1,38}$ = 5.77, p = 0.021, group-by-time interaction effect $F_{1,38}$ = 1.45, p = 0.24; gain vs loss in WT: effect of group $F_{1,20}$ = 0.42, p = 0.52, effect of time $F_{1,20}$ = 0.01, p = 0.94, group-by-time interaction effect $F_{1,20}$ = 5.95, p = 0.024; gain vs loss in APPPS1: effect of group $F_{1,56}$ = 1.35, p = 0.25, effect of time F1,56 = 1.26, p = 0.27, group-by-time interaction effect $F_{1,56}$ = 8.59, p = 0.005, two-way repeated measures ANOVA). **(d)** Fraction of intermediately active neurons turning highly active at week 2 (30 out of 309 intermediately active in WT and 200 out of 951 intermediately active in APPPS1 mice, $p < 10^{-5}$, Fisher's exact test). **(e)** Activity gain of novel highly active neurons at week 2 (p = 0.1, Mann-Whitney U test, data are median +/- 95% CI). **(f)** Fraction of intermediately active neurons turning highly active from week 2- 4 (26 out of 355 intermediately active in WT and 180 out of 980 intermediately active in APPPS1 mice, $p < 10^{-6}$, Fisher's exact test). **(g)** Activity gain from week 2-4 of those novel highly active neurons (p = 0.82, Mann-Whitney U test, data are median +/- 95% confidence interval). Data are mean +/- SEM; * P < 0.05, ** P < 0.01, *** P < 0.001

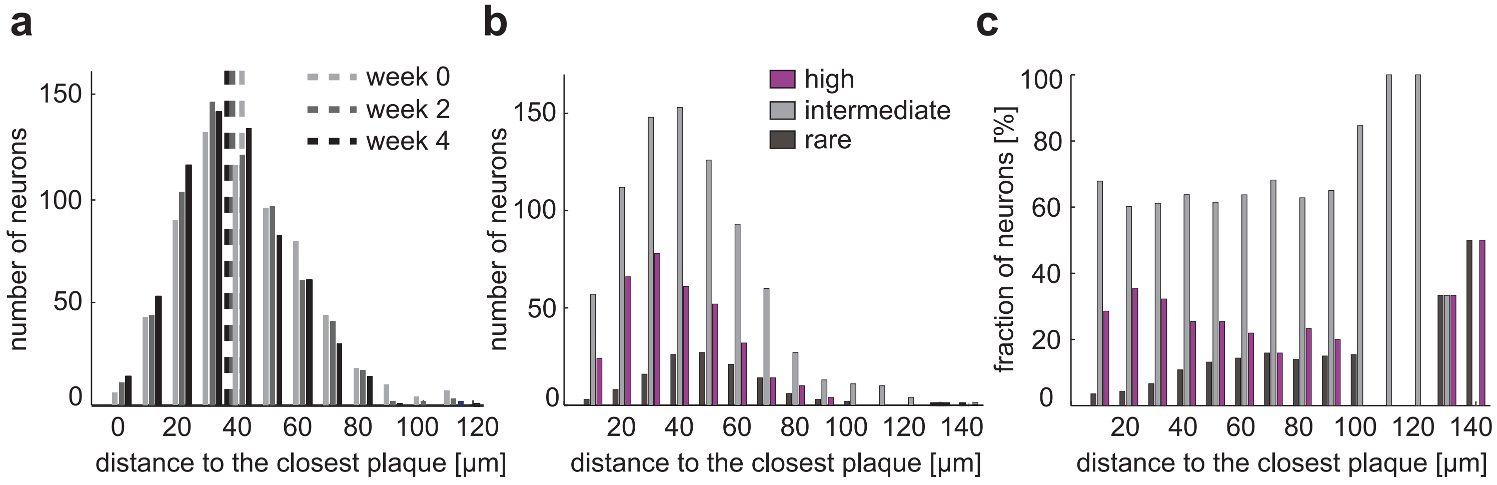

Supplementary Fig. 6. Distribution of plaque distances. (a) Distribution of the distances between neurons and their closest plaque at the three imaging time points (median of each time point shown as dashed line). **(b)** Distribution of the absolute number of neurons within the three neuronal activity categories at the first imaging time point as a function of plaque distance. **(c)** Distribution of the relative fraction of neurons for the three activity categories at the first imaging time point as a function of plaque distance (data is based on pooled neurons from 15 experiments). Of note, beyond 100 µm very few cells were found, which affects the proportional abundance of the three categories.

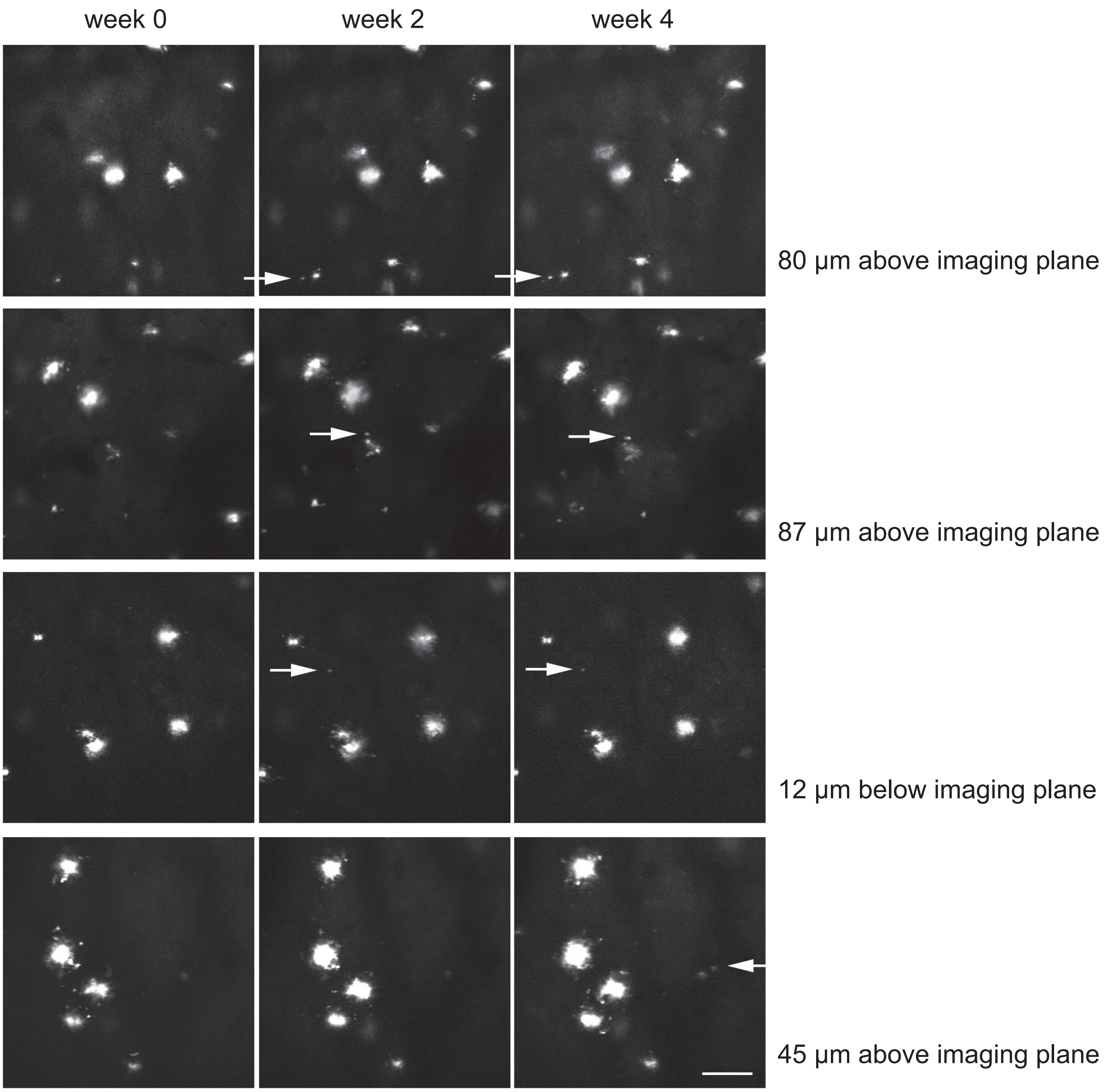

Supplementary Fig. 7. Novel amyloid plaques form close to existing ones.
Representative examples of averaged projections of ~20-30 µm are shown for each imaging time point. Novel plaques (marked by white arrows at the first time of appearance) are small and primarily occur close to pre-existing plaques. Distance of the novel plaque in z from the imaging plane is given on the right hand side. Scale bar 50 µm.

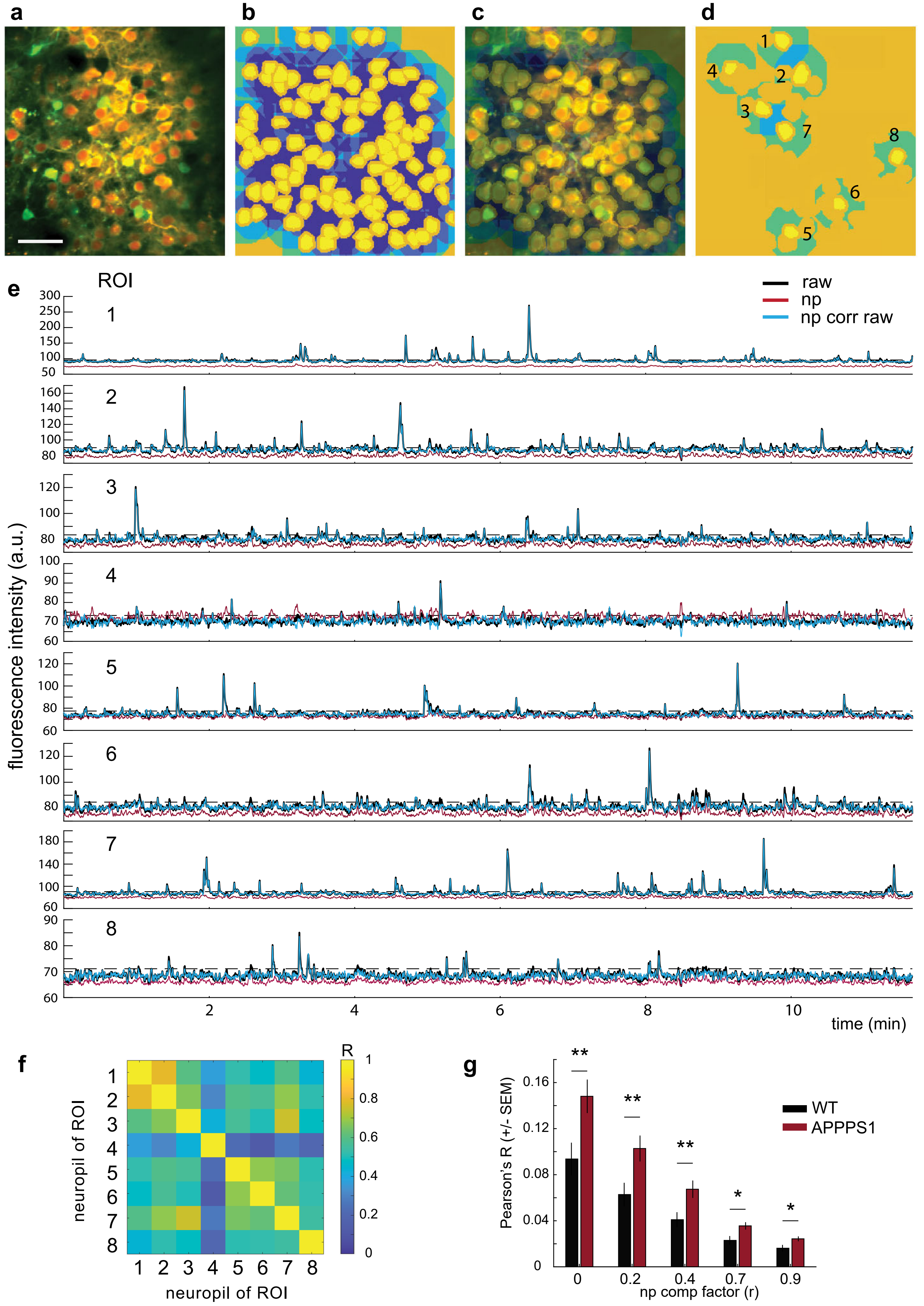
a
b
c
d
1
2
3
4
5
6
7
8
e
ROI
raw
np
np corr raw
fluorescence intensity (a.u.)
time (min)
f
R
neuropil of ROI
neuropil of ROI
g
Pearson's R (+/- SEM)
np comp factor (r)
WT
APPPS1
**
**
**
*
*

Supplementary Fig. 8. Impact of neuropil compensation on pairwise activity correlation.
(a) Average projection of time scan of example field of view (fov; red - expression of mRuby2, green - expression of GCaMP6). **(b)** Map of individual ROIs (yellow) along with their respective surrounding areas used for neuropil compensation (turquious to dark blue). The area right in contact with the soma was excluded from the neuropil assessment (orange ring) as were other cell bodies. **(c)** Average projection of the FOV (shown in a) superimposed by the ROI mask (shown in b). **(d)** Representative ROIs (yellow) together with their surrounding area used to estimate the fluorescence of the adjacent neuropil (turquoise) are shown. 'Defects' in the otherwise ringlike neuropil area correspond to other ROIs, which were excluded from it. **(e)** Absolute values of fluorescence intensities of the example ROIs (in d) are shown (black trace - raw data, red - neuropil, blue - raw data compensated for neuropil contamination using a factor of 0.7. Dashed lines depict threshold for binarization of data applied prior to correlation analysis. **(f)** Correlation matrix of neuropil fluorescence of the example ROIs shown in (d). Median correlation is 0.52 for the here shown 8 areas. **(g)** More stringent neuropil compensation reduces pairwise neuronal activity correlation. However, neuronal correlations in APPPS1 were consistently higher compared to WT mice, independent from the neuropil compensation factor (shown are the average Pearson's correlation factors across all 3 time points analysed for each fov applying different compensation factors (r); $r = 0$: $p = 0.003$, $r = 0.2$: $p = 0.004$, $r = 0.4$, $p = 0.005$, $r = 0.7$, $p = 0.013$, $r = 0.9$, $p = 0.028$, all student's t-test, * $p < 0.05$, ** $p < 0.01$). Scale bar (a) 50 µm.

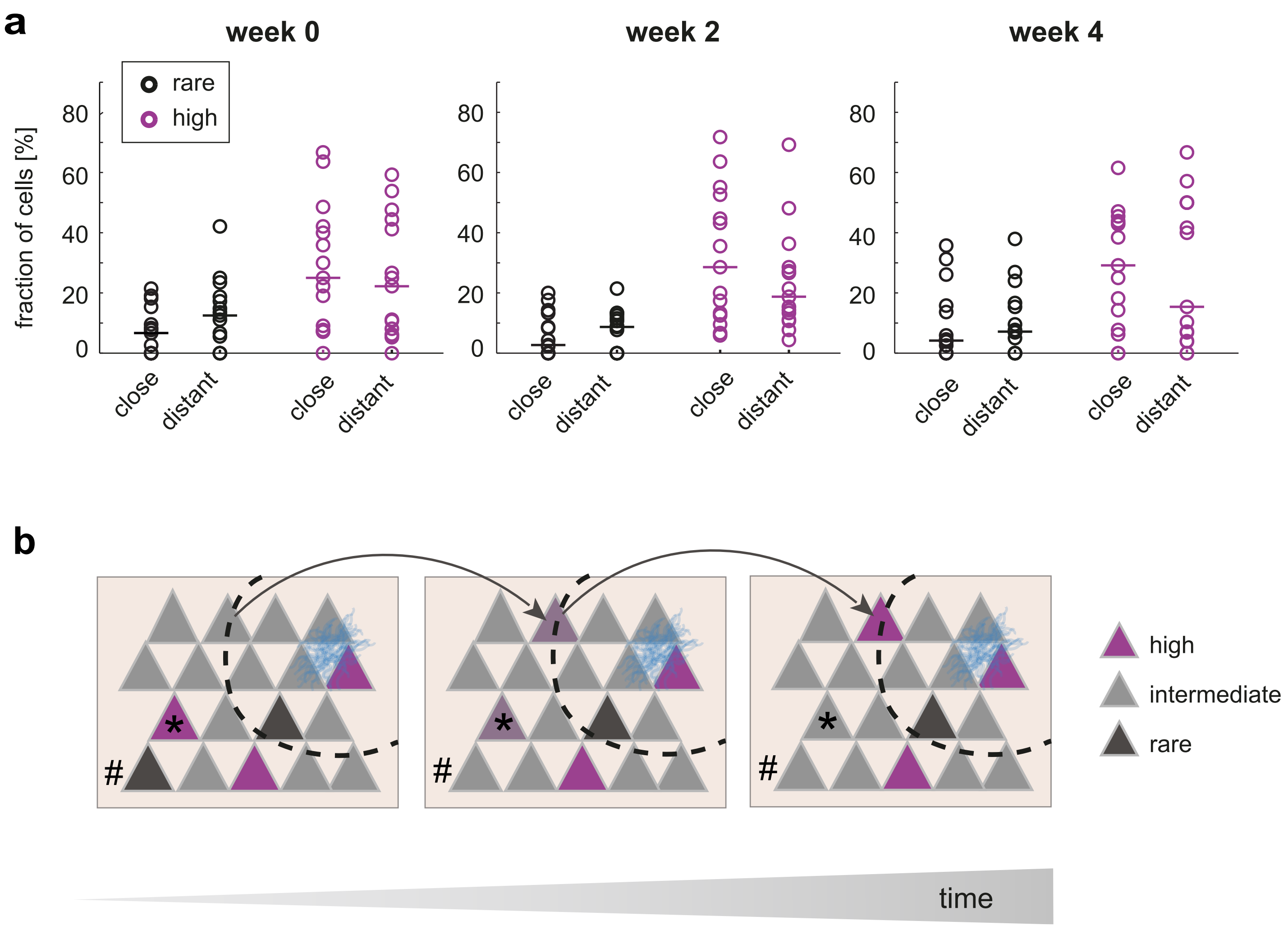

Supplementary Fig. 9. Fractions of rarely and highly active neurons close and distant from amyloid plaques. (a) The fraction of highly and rarely active neurons did not differ between close and distant neurons at the level of individual experiments (week 0: fraction rarely active close vs. distant p = 0.25, fraction highly active close vs. distant p = 0.63; week 2: fraction rarely active close vs. distant p = 0.78, highly active close vs. distant p = 0.38; week 4: fraction rarely active neurons close vs. distant p = 0.63, fraction highly active close vs. distant p = 0.93; horizontal line denotes the median. close n = 15 experiments, distant n = 15 experiments, Mann-Whitney U test). **(b)** Scheme illustrating key aspects in the evolution of aberrant neuronal activity during the early phase of AD pathology. Neuronal activity levels in cortex are already altered during the early phase of amyloid plaque deposition (amyloid plaque depicted in blue), resulting in a higher fraction of highly active neurons (magenta). Activity levels remain largely constant over extended periods of time. A proportion of intermediately active neurons (gray) slowly increase their activity thereby turning into highly active neurons over time (example marked by arrow). Distant from plaques (dashed line) aberrant activity (both rarely (#) and highly (*) active cells) is less likely to persist compared to the immediate plaque vicinity.
