# Supplementary material for: Long-term dynamics of aberrant neuronal activity in awake Alzheimer’s disease transgenic mice
Source: Commun Biol. 2021 Dec 7;4:1368. doi: 10.1038/s42003-021-02884-7 (PMC8651654; doi:10.1038/s42003-021-02884-7)
Supplement: Supplementary file 2 — Description of Additional Supplementary Files [file 42003_2021_2884_MOESM2_ESM.pdf]

Description of Additional Supplementary Files

File name: Supplementary Data 1
Description: Average neuronal activity over time.

File name: Supplementary Data 2
Description: Soluble and insoluble amyloid beta concentrations.

File name: Supplementary Data 3
Description: Activity change of individual neurons over time.

File name: Supplementary Data 4
Description: Similarity index of activity levels.

File name: Supplementary Data 5
Description: Activity category of individual neurons in WT mice.

File name: Supplementary Data 6
Description: Activity category of individual neurons in APPPS1 mice.

File name: Supplementary Data 7
Description: Activity change highly active neurons.

File name: Supplementary Data 8
Description: Activity change intermediately active neurons.

File name: Supplementary Data 9
Description: Activity change rarely active neurons.

File name: Supplementary Data 10
Description: Reoccurrence rate of activity levels.

File name: Supplementary Data 11
Description: Fraction of stable activity category for neurons close and distant to amyloid plaques.

File name: Supplementary Data 12
Description: Activity change as function of plaque distance.

File name: Supplementary Data 13
Description: Pairwise activity correlations.

File name: Supplementary Data 14
Description: Pairwise activity correlations during stationary and whisking epochs.
